# Structural determinants of DNA recognition by the NO sensor NsrR and related Rrf2-type [FeS]-transcription factors

**DOI:** 10.1038/s42003-022-03745-7

**Published:** 2022-07-30

**Authors:** Roman Rohac, Jason C. Crack, Eve de Rosny, Océane Gigarel, Nick E. Le Brun, Juan C. Fontecilla-Camps, Anne Volbeda

**Affiliations:** 1 Univ. Grenoble Alpes, CEA, CNRS, Institut de Biologie Structurale, Metalloproteins Unit, F-38000 Grenoble, France; 2 Centre for Molecular and Structural Biochemistry, School of Chemistry, University of East Anglia, Norwich Research Park, Norwich, NR4 7TJ UK

**Keywords:** X-ray crystallography, DNA, Metalloproteins, Transcription

## Abstract

Several transcription factors of the Rrf2 family use an iron-sulfur cluster to regulate DNA binding through effectors such as nitric oxide (NO), cellular redox status and iron levels. [4Fe-4S]-NsrR from *Streptomyces coelicolor* (*Sc*NsrR) modulates expression of three different genes via reaction and complex formation with variable amounts of NO, which results in detoxification of this gas. Here, we report the crystal structure of *Sc*NsrR complexed with an *hmpA1* gene operator fragment and compare it with those previously reported for [2Fe-2S]-RsrR/*rsrR* and apo-IscR/*hyA* complexes. Important structural differences reside in the variation of the DNA minor and major groove widths. In addition, different DNA curvatures and different interactions with the protein sensors are observed. We also report studies of NsrR binding to four *hmpA1* variants, which indicate that flexibility in the central region is not a key binding determinant. Our study explores the promotor binding specificities of three closely related transcriptional regulators.

## Abstract (web summary)

The crystal structure of the iron-sulfur protein NsrR from *Streptomyces coelicolor* bound to a gene operator fragment is reported and compared with other structures, giving insight into the structural determinants of DNA recognition by the NO sensor.

## Introduction

All microorganisms need to rapidly sense environmental changes and to respond to them in order to survive, adapt and reproduce. This response is mainly mediated by transcription factors (TFs) that regulate the expression of relevant proteins by either blocking or favoring the activity of RNA polymerases at specific gene sites^1,2^. Besides their cognate DNA-binding site region, TFs generally have a domain that recognizes effectors, such as other proteins or small ligands. Members of the CRP-FNR and Rrf2 families of dimeric microbial TFs can use the gas-binding affinity, redox properties and intrinsic lability of iron-sulfur clusters to modulate their DNA binding^3–7^. The well-studied Fumarate Nitrate Reductase regulator (FNR) uses a [4Fe-4S] cluster to sense O_2_ levels and to regulate a dimer ↔ monomer transition mediated by cluster disassembly and a metastable protein interface^8^. Only the dimer binds to specific DNA sites^9^. The Rrf2 family was first described in *Desulfovibrio vulgaris* Hildenborough^10^ and the three-dimensional structure of the cysteine metabolic regulator CymR from *Bacillus subtilis* (*Bs*), which does not bind an iron-sulfur cluster, was the first to be reported for a member of this family^11^. Structural and functional studies of the related *Staphylococcus aureus* (*Sa*) CymR revealed a sensing mechanism in which DNA binding is lost under oxidative stress conditions after oxidation of a surface cysteine thiol^12^. *Bs*CymR binds to DNA after forming a complex with O-acetyl-thiol-lyase only when cysteine is present^13^.

Members of the Rrf2 family that coordinate iron-sulfur clusters use them to sense various effectors and regulate gene expression to enable adaptation, maintenance of homeostasis and cell protection. Characterized cluster-binding TFs include: IscR from *Escherichia coli (Ec)* and *Thermincola potens* (*Tp*) that sense cell iron-sulfur cluster levels and oxidative stress^6,14^; RsrR, a redox-sensitive response regulator from the soil bacterium *Streptomyces venezuelae* (*Sv*)^5^; *Rhizobium leginosarum* (*Rl*) RirA that uses a labile [4Fe-4S] cluster to regulate cellular iron concentrations^15^; and NsrR, a regulator of nitric oxide (NO)-induced stress in many bacterial species^4,16^. The regulation of their DNA binding capabilities depends on protein conformational changes caused by the status of the cluster or its absence. The first crystal structures of an iron-sulfur sensor of the Rrf2 family were those of the apo-form of *Ec*- and *Tp*-IscR^17,18^. More relevant to the work reported here, the structure of apo-*Ec*IscR in complex with a 29 base pair (bp) fragment of the hydrogenase-1 *hyA* operator was also solved^17^. The structure of [2Fe-2S]-IscR, which recognizes different operator sequences^19^, has not yet been determined, either alone or in complex with DNA.

We have reported the crystal structures of [2Fe-2S]-*Sv*RsrR alone^20^ and in complex with cognate DNA, the latter at medium resolution^21^. Using a combined approach of structural and mass spectrometric studies and molecular dynamics calculations, we were able to show how the one-electron cluster reduction of [2Fe-2S]-*Sv*RsrR triggers protonation of a histidine residue and the burial of a tryptophan side chain, causing conformational changes that lead to DNA dissociation^21^. Our groups have also reported the holo- and apo- crystal structures of NsrR from the antibiotic-producing bacterium *Streptomyces coelicolor* (*Sc*)^22^. The principal regulatory target of this TF is, in most organisms, the *hmp* gene, which encodes a flavohemoglobin that converts NO to nitrate (NO_3_^−^) or nitrous oxide (N_2_O) under aerobic or anaerobic conditions, respectively^23,24^.

*Sc*NsrR is so far the only NsrR to have been characterized in significant detail^22,24–28^. Its regulon is small, enabling a detailed investigation of the relative DNA binding properties of [4Fe-4S]-*Sc*NsrR to each identified operator: *hmpA1, hmpA2*, and *nsrR*, showing a 23-base pair (bp) consensus recognition site. Tightest binding was observed for *hmpA1*, followed by *nsrR* and *hmpA2*.^25^ In the presence of NO, the *Sc*NsrR [4Fe-4S] cluster is rapidly lost, causing the dissociation of the protein-DNA complex. Binding of *hmpA2* was found to be the most sensitive to NO, followed by *hmpA1* and *nsrR*^24^. Apo-*Sc*NsrR (or [2Fe-2S]-*Sc*NsrR, which could be generated and stabilized upon O_2_ exposure in the presence of β-mercaptoethanol)^29^ did not bind DNA with significant affinity^25^. In some bacteria, such as *Ec* and *Salmonella enterica* serovar Typhimurium, many more genes are under NsrR control, suggesting a broader regulatory role for NsrR in these microorganisms^30,31^.

A comparison of the three-dimensional structures of holo [4Fe-4S]-*Sc*NsrR and apo-*Sc*NsrR furnished a first model to understand how NO binding to the iron-sulfur cluster, and the resulting conformational changes, control the configuration of the protein DNA-binding surface^22^. Here, we report the 3.0 Å resolution X-ray structure of *Sc*NsrR complexed with a 23-bp *hmpA1* operator fragment and use it, alongside the structures referred to above, to establish what determines the DNA binding specificity among the three structurally-related iron-sulfur Rrf2 family proteins IscR, RsrR and NsrR. DNA recognition is generally considered to depend on either a direct readout of the nucleobase sequence, a readout of a sequence-dependent DNA phosphate backbone shape, or a combination thereof^32,33^. Related to the recognition process is the flexibility of the DNA operator regions and their propensity to undergo conformational changes upon protein binding. In a classical study^34^, a crystalline B-DNA dodecamer went from an almost straight double helix structure to a bent one as the temperature was changed from 7 °C to 20 °C. DNA flexibility has also been shown by solution NMR studies: the ^31^P chemical shifts of double stranded (ds) B-DNA fragments vary as a function of the sequence-dependent ratio of B_I_ and B_II_ backbone states. These are in a dynamic conformational equilibrium, with B_I_ being the most commonly observed in regular B-DNA^35^. We have explored these aspects by comparing the remarkably different DNA binding modes of [4Fe-4S]-*Sc*NsrR (this work), apo-IscR^17^ and [2Fe-2S]-RsrR^21^ in their respective crystal structures. In addition, we have tested four modified oligonucleotides sequences between two recognition sites of the NsrR regulator in order to explore the effect of DNA flexibility on its binding. This analysis allows us to propose a series of structural features that determine specific DNA binding within a class of closely related proteins.

## Results

### Structure of the [4Fe-4S]-*Sc*NsrR-*hmpA1* operator complex

**Fig. 1:**
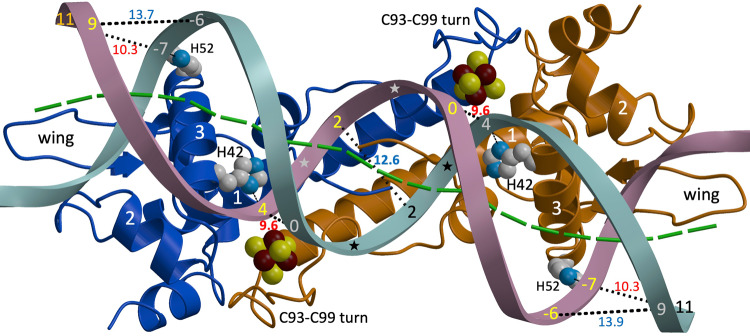
Top view of the *Sc*NsrR/*hmpA1* operator complex. NsrR subunits Cα tracings are depicted in blue and gold with ribbons indicating α-helices and arrows β-strands. DNA strands carry nucleotide numbers and are shown as cyan and burgundy ribbons, with stars marking four B_II_ backbone conformations in the central region. Cluster atoms and histidine side chains forming hydrogen bonds to phosphate groups are depicted as spheres (Fe: red-brown, S: yellow, N: blue and C: gray). The first three helices and the labeled wing loop of each protein subunit interact with the DNA. Minimal and maximal P-to-P distances between phosphate groups across the minor groove are indicated with dotted lines and given in Å. The green dashed line follows the bp origins of the DNA double helix as determined with the program DSSR^37^.

Table 1 shows the statistics of the 3.0 Å resolution structure of [4Fe-4S]-*Sc*NsrR complexed to the 23-bp *hmpA1* operator fragment. High atomic temperature (B) factors indicate significant disorder, in agreement with the flexible and dynamic structure previously observed for the noncomplexed protein structure^22^. Both the operator fragment and the bound protein dimer are about 80 Å long (Fig. 1). The buried surface area (BSA) of the protein/DNA complex is 4222 Å^2^, comparable to the BSA of 4981 Å^2^ of the NsrR dimer but much larger than the 1996 Å^2^ buried between the two strands of the DNA helical fragment. The BSA at the protein/DNA interface of the *Ec*IscR/*hyA* complex^17^ is 3780 Å^2^. This relatively low value is explained by the fact that some of the amino acids of *Ec*IscR facing the central region of the *hyA* operator were not resolved and therefore not included in the structure. A higher value of 4490 Å^2^ is obtained for the *Sv*RsrR/*rsrR* complex^21^ because the larger 39 bp *rsrR* operator fragment covers the protein sensor more extensively than the 23 bp *hmpA1* one does.

**Table 1:** X-ray data collection and refinement statistics.

X-ray data		Refinement	
Space group	P6_5_	Resolution (Å)	51.45–3.00
Cell dimensions		Reflections (work/free)	13678/716
a = b, c (Å)	118.8 89.5	R_work_/R_free_	22.0/23.9
α = β, γ (°)	90 120	Number of atoms	3114
Resolution (Å)^a^	67.54–3.0 (3.18–3.0)	Protein	2151
best in h k plane	3.14	DNA	943
best along l axis	3.00	[4Fe-4S] cluster	16
R_sym_ (%)	5.9 (194.5)	Water	4
Mean(I/σ(I))	12.6 (1.0)	Average B factors (Å^2^)	
CC(1/2)	0.999 (0.516)	Protein	134.2
Completeness (%)	99.8 (100.0)	DNA	181.4
Redundancy	6.3 (6.7)	[4Fe-4S] cluster	110.7
		Water	118.1
		R.m.s. deviations	
		Bond lengths (Å)	0.003
		Bond angles (°)	0.70

^a^Values within parentheses denote the highest resolution shell.

Residues 59–64 of the *Sc*NsrR dimer wings, which are disordered in the free protein^22^, are partially resolved in the complex where they interact with the ends of the *hmpA1* operator fragment (Fig. 1). Other DNA binding regions include the N-termini of α-helices 1 and 2 and the side of the third one, which corresponds to the regulatory helix (RH) of a helix-turn-helix (HTH) motif. Conversely, the C93-C99 turn, which we previously considered as a potential additional contact interface^22^, does not directly interact with the DNA. Compared to many other proteins with a winged (w) HTH motif^36^, the DNA binding region of *Sc*NsrR contains an additional preceding α-helix and, consequently, can be better described as an H-wHTH motif.

#### Structure of the DNA hmpA1 fragment

**Fig. 2:**
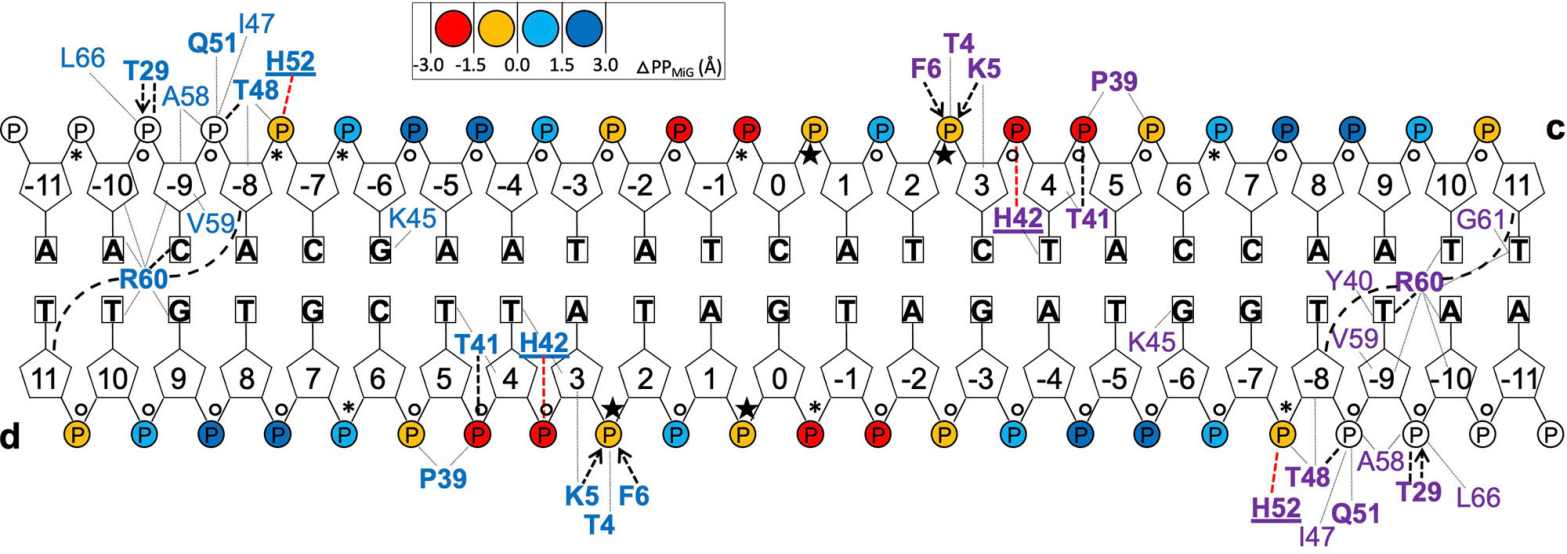
Cartoon of the *Sc*NsrR-*hmpA1* complex. Blue- and purple-labeled residues belong to different NsrR subunits. Nucleotides are numbered from 0 at the center of the figure. Contacts with distances (d) < 3.3 Å from *hmpA1* strands **c** and **d** to NsrR residues depicted in bold are shown by black dashed lines for H-bonds, by dashed arrows if a main chain N atom is involved and by red dashed lines for possible salt bridges with His42 and His52. Thin lines depict remaining contacts with d < 3.8 Å, including those for disordered NsrR residues (see also Table S1). Circles depicting phosphate (P) groups are colored according to the corresponding minimal P-to-P distance across the MiG relative to its average value of 11.7 Å in regular B-DNA (ΔPP_MiG_). Small circles and black stars indicate phosphate torsion angles in the B_I_ and B_II_ backbone conformation, respectively; asterisks highlight those with an intermediate conformation.

**Fig. 3:**
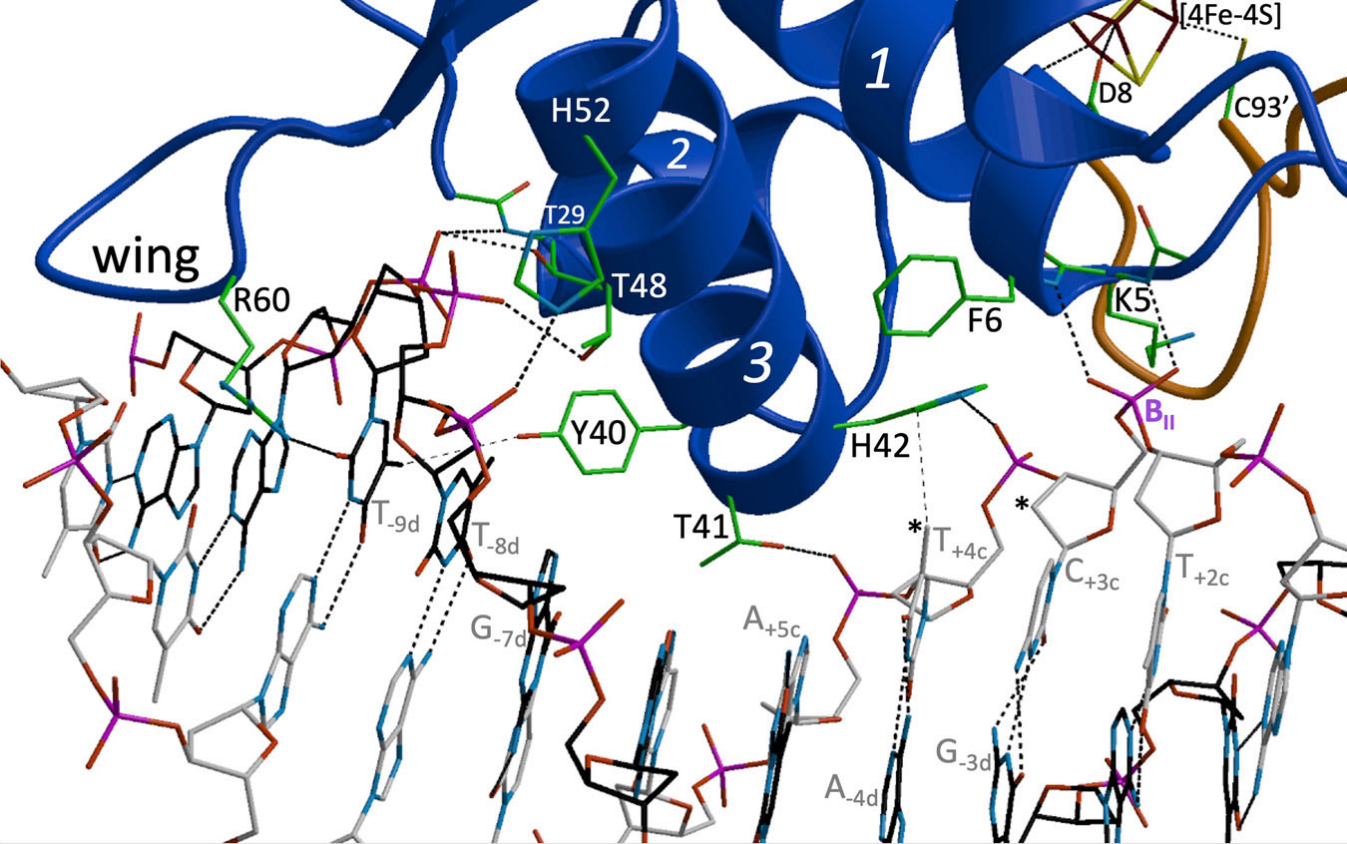
Close up view of the *Sc*NsrR/*hmpA1* interface. Cα traces of the A and B subunits are depicted in gold and blue, respectively; ribbons are used to depict α-helices. DNA double helix bonds and contacting NsrR residues are represented with sticks. Hydrogen bonds and salt bridges are indicated with thick dashed lines; two van der Waals contacts are shown as thin dashed lines. The asterisks (*) label the T_+4c_-methyl group and the C_+3c_-C2’ atom. Atom color codes: Fe brown-red, S yellow, P pink, O red, N blue and C green, black or gray. For stereo versions of this figure see Fig. S3a, b.

The 23 bp *hmpA1* operator fragment has a B-DNA conformation. The double helix runs almost straight through the central 11 bp region and bends at its extremes around α*-*helix 3 of each of the NsrR subunits (Fig. 1). The distance between phosphate P atoms across the minor groove (MiG) decreases from 12.6 Å at the center of the fragment to 9.6 Å between nucleotides 0 and + 4 of the other strand. It increases again to about 13.8 Å between nucleotides +9 and −6 before narrowing to 10.3 Å between nucleotides + 11 and −7. Minimal MiG distances are observed for the + 4 and −7 phosphates, which respectively interact with the His42 and His52 imidazole groups. An analysis of torsion angles with the program DSSR^37^ (Fig. S1) shows that 32 of the 44 phosphate groups in the bound 23 bp *hmpA1* operator fragment (excluding the 5’-terminal ones) are in the B_I_ conformation. Conversely, the phosphates bound to the C3’ atom of the 0 and + 2 nucleotides are in the B_II_ conformation (see also Fig. S2), while the remaining ones are in a conformation intermediate between B_I_ and B_II_ (Fig. 2). The reorientation of the T_+2_ 3’-phosphates from B_I_ to B_II_ conformations causes propeller twists of −19.0° for bp C_+3c_/G_-3d_ (Figs. 3 and S3) and −15.9° for bp T_-3c_/A_+3d_.

#### Protein-DNA interactions

Most of the *Sc*NsrR contacts are made with the phosphate backbone of the *hmpA1* operator (Figs. 2, 3 and Table S1). The His42-Nδ1 and His52-Nε2 atoms interact with the respective 5^’^-phosphate groups of nucleotides +4 and −7 in opposite strands (Figs. 1–3). These interactions could be salt bridges if the Nδ1 and Nε2 atoms of the imidazole groups are protonated. The side chains of Arg2, Lys5 and Lys45, present at the contact interface, are disordered (Fig. S4), as they were in the 1.95 Å resolution structure of non-complexed [4Fe-4S]-*Sc*NsrR^22^. However, these basic residues could still interact with the negatively charged DNA phosphate backbone (Fig. S5). The positive dipole moments at the N-termini of α-helices 1 and 2 provide additional electrostatic stabilization via H-bonds from: (i) Lys5 main chain N atom to OP1 and Phe6-N to OP2 of the 5^’^-phosphate group of the +3 nucleotide, which is in a B_II_ conformation (the non-complexed *Sc*NsrR structure has a bound sulfate at this position, see Fig. S6); (ii) Thr29 main chain N atom to the 5^’^-phosphate of the −9 nucleotide in the opposite strand, which is also hydrogen bonded to Thr29-Oγ1. The Thr41 and Thr48 Oγ1 atoms of α-helix 3 make similar H-bonds to the respective 5^’^-phosphates of the +5 and −8 nucleotides in opposite strands.

There are only a few specific interactions between *Sc*NsrR amino acid side chains and the nucleobases of the *hmpA1* operator (Fig. 3 and Table S1). In the major groove (MaG), Lys45 could establish H-bonds to ring atoms of G_−6_ and surrounding bases. However, its weak electron density (Fig. S4) makes a definite assignment difficult. The 58–61 and 66 wing residues bind edge-on to the MiG (see also Fig. S3b), with Arg60 making the most contacts to the DNA. These include a H-bond between its guanidinium group and the O2 atom of the pyrimidine base at the −9 position and van der Waals (vdW) contacts with T_+10_-O2 and G_+9d_-N2. Gly61 makes vdW contacts with T_+11_-O2. Base-specific vdW contacts are observed in the MaG from Thr41_A_ (4 atoms), His42-Cδ2 and Tyr40_B_-Oη to the C7 atoms of T_+5d_, T_+4_ and T_-9d_, respectively (Table S1). Additional resolved interactions with deoxyribose atoms involve the RH Thr41 and Thr48 and the wing Arg60.

### Comparison of the protein-DNA complex with DNA-free *Sc*NsrR structures

**Fig. 4:**
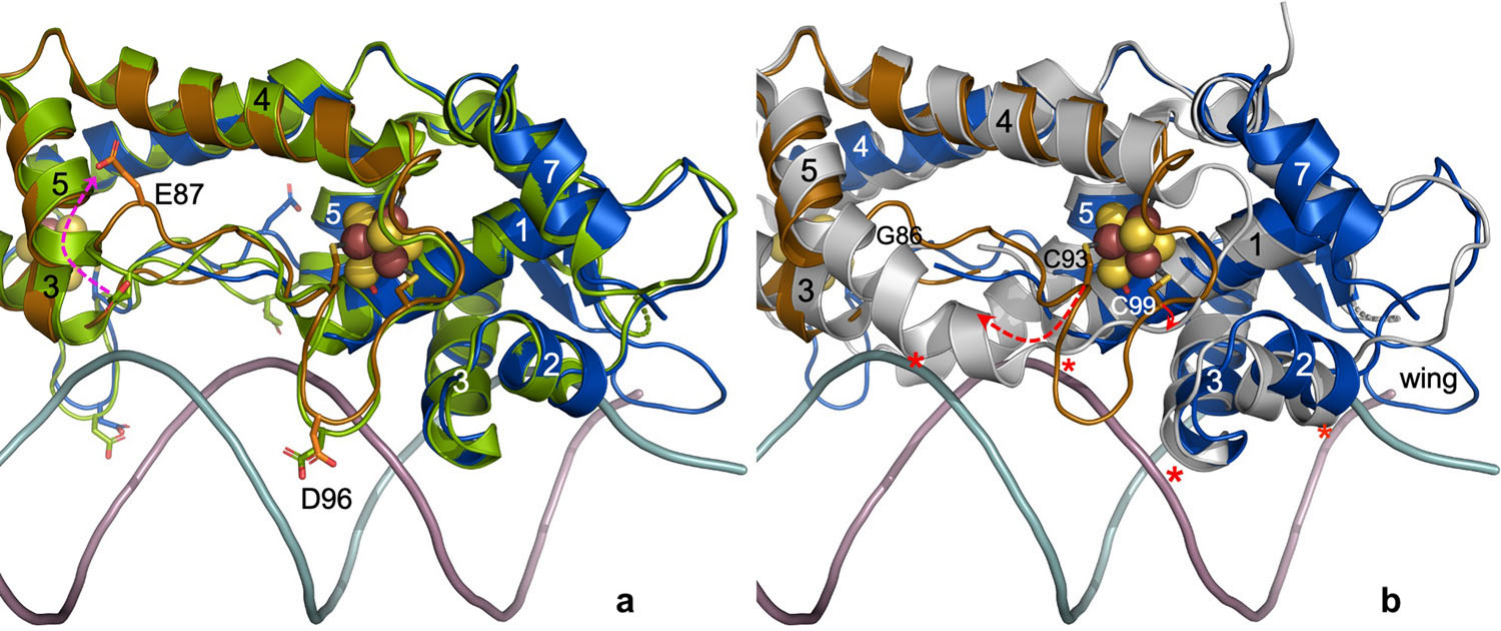
Comparison of [4Fe-4S]-*Sc*NsrR/*hmpA1* complex with DNA-free structures. **a** Structural superposition of the complex to [4Fe-4S]-*Sc*NsrR (green, cluster not shown). **b** Superposition to apo-3CA-*Sc*NsrR (gray). The complex is colored as in Fig. 1 and selected amino acid residues are highlighted. Red arrows indicate movements observed for residues 87, 93 and 99. Regions of the apo structure that would collide with *hmpA1* are indicated by red asterisks. Atom color codes are as in Fig. 3. Rotated stereo zooms of the same superpositions are given in Fig. S6 and S8.

A cavity analysis indicates that the putative access path for NO that runs along the cluster ligand Asp8, previously observed in [4Fe-4S]-*Sc*NsrR^22^, is also present in its complex with the 23 bp *hmpA1* operator fragment (Fig. S7). The most important structural difference in the latter is a rearrangement of the region following α-helix 5 (Fig. 4a) caused by the electrostatic repulsion between the negatively charged Glu87 and the phosphate backbone. A similar effect could have provoked a small shift of Asp96 away from the DNA (see also Fig. S6). A much larger structural rearrangement of the 87–105 region is observed in the apo-3CA-*Sc*NsrR variant where the cluster-coordinating Cys93, Cys99 and Cys105 were substituted by Ala (Figs. 4b and S8). In this variant^22^, helix 5 is kinked at Gly86 and extends to Gly95. Consequently, the C93-C99 loop, which in the [4Fe-4S]-*Sc*NsrR-DNA complex faces the MiG of the *hmpA1* central region (Fig. 4a), adopts an entirely different conformation. Because α-helices 1, 2 and 3 are rearranged in the apo structure, its complex with *hmpA1* DNA is not stereochemically possible as it would involve several severe clashes with the N-terminal regions of these helices (Fig. 4b).

### Comparison with other sensors of the Rrf2 family

**Fig. 5:**
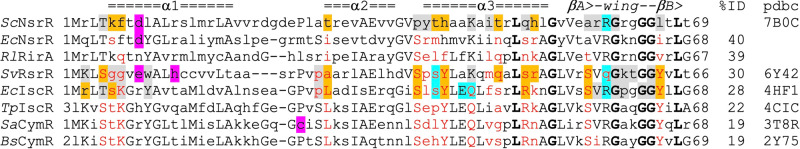
H-wHTH motif sequence alignment of eight selected Rrf2 family members. Numbered α-helices (H) and labeled wing β-strands are shown on top for *Sc*NsrR; residues conserved in at least four sequences are indicated with capitals and given in bold when they are invariant. Percentages of sequence identity with *Sc*NsrR and Protein Data Bank codes for the structures used here are given in the %ID and pdbc columns, respectively. Residues with H-bond or salt bridge distances ≤ 3.3 Å to phosphate groups in the *Sc*NsrR/*hmpA1*, *Sv*RsrR/*rsrR* and *Ec*IscR/*hyA* structures are highlighted in orange and those that form H-bonds to nucleobases are depicted in turquoise; additional residues within 3.8 Å of the bound DNA fragments are highlighted in gray and those that are different from *Sc*NsrR are shown in red. Cluster binding residues in *Sc*NsrR and *Sv*RsrR and the oxidation-sensitive cysteine of *Sa*CymR are highlighted in purple. Abbreviations for different bacteria are defined in the text. *Tp*, *Sa*, and *Bs* are Gram-positive bacteria, all the others are Gram-negative.

The homologous *E. coli* NO sensor displays several amino acid sequence differences with *Sc*NsrR in the DNA contacting region (Fig. 5). For example, *Sc*NsrR Thr29, Thr41 and Thr48, which are hydrogen-bonded to phosphate groups of *hmpA1*, are neither conserved in *Ec*NsrR, nor replaced by Ser, which would allow a similar interaction. In addition, the phosphate-binding His52 is substituted by Arg. These dissimilarities could explain why *Sc*NsrR does not display significant affinity for *Ec*NsrR-specific DNA sequences^25^. The above mentioned His and Thr residues are also not conserved in *Sv*RsrR or *Ec*IscR, the other two Rrf2 family iron-sulfur cluster binding sensors with known DNA complex structures, although Thr41 is replaced here by Ser. His42, the other *Sc*NsrR residue that possibly makes a salt bridge with the phosphate backbone, is replaced by Tyr in those sensors. Amino acid sequence comparisons, including *Rl*RirA and *Sa* and *Bs*CymR, reveal a conserved Lx_3_Gx_6_Gx_2_GGx_2_L motif (Fig. 5) that spans the C-terminal region of helix 3 and the wing region. The comparison shows that most of the *Sc*NsrR DNA contacting residues are not conserved in the eight Rrf2 family members. This indicates, as might be expected, that the different binding modes observed for these sensor/DNA complexes result from a combination of specific amino acid (Fig. 5) and nucleotide (Table 2) differences. A detailed comparison of the *Sc*NsrR/*hmpA1* and *Ec*IscR/*hyA* complex structures reveals a small number of base-specific interactions (Table S1). Modelling different operator sequences on the structures of *hmpA1* and *hyA* produces vdW collisions with the respective *Sc*NsrR and *Ec*IscR protein structures (shown in ***bold italics*** in Table 2).

**Table 2:** NsrR and IscR base specificity for different operator fragments*.

*base*	*hmpA1*	d (Å)	*Sc*NsrR	*hyA*	d (Å)	*rsrR*	d (Å)	*hyA*	d (Å)	*Ec*IsrR	*hmpA1*	d (Å)	*rsrR*	d (Å)
−10c	A-N3	3.0	R_60A_O	A-N3	3.0	***C-O2***	***2.8***							
−10d	A-N3	3.0	R_60B_O	A-N3	3.0	***C-O2***	***2.8***							
−8c								C-N4	2.7	E_43B_Oε2	A-N7	3.1	C-N4	2.7
−8d								C-N4	2.8	E_43A_Oε2	***T-C7***	***2.5***	C-N4	2.8
+4c	T-C7	3.5	H_42B_Cδ2	T-C7	3.6	G		T-O4	3.2	Q_44A_Nε2	T-O4	3.2	G	
+4d	T-C7	3.6	H_42A_Cδ2	T-C7	3.6	A		T-O4	3.0	Q_44B_Nε2	T-O4	3.0	A	
+5c	A-C8	3.6	T_41B_Oγ1	A-C8	3.6	T-C7	2.9	A-N7	2.9	S_40A_Oγ	A-N7	2.9	***T-C7***	***1.8***
+5d	T-C7	3.6	T_41A_Cβ	G-C8	3.9	T-C7	3.6	G-N7	2.7	S_40B_Oγ	***T-C7***	***1.8***	***T-C7***	***1.8***
+5c								A-N7	3.4	S_40A_Cβ			***T-C7***	***1.6***
+5d								G-N7	3.2	S_40B_Cβ	***T-C7***	***1.5***	***T-C7***	***1.5***
+6c	C		T_41B_Cγ2	***T-C7***	***2.7***	C								
+6d	C		T_41A_Cγ2	***T-C7***	***2.7***	C								
+10c	T-O2	3.4	R_60B_Cδ	T-O2	3.4	***G-N2***	***2.5***	T-O2	3.3	R_59A_Nη2	T-O2	3.3	G-N3	3.4
+10d	T-O2	3.3	R_60A_Cδ	T-O2	3.3	***G-N2***	***2.2***	T-O2	3.5	R_59B_Nη2	T-O2	3.5	G-N3	3.6

^*^These are modeled on the known structures of *hmpA1* and *hyA* complexed with *Sc*NsrR and *Ec*IscR. For repulsive interactions the contact distance (d) and corresponding base substitution are in bold italics.

#### DNA operator fragments conformations

**Fig. 6:**
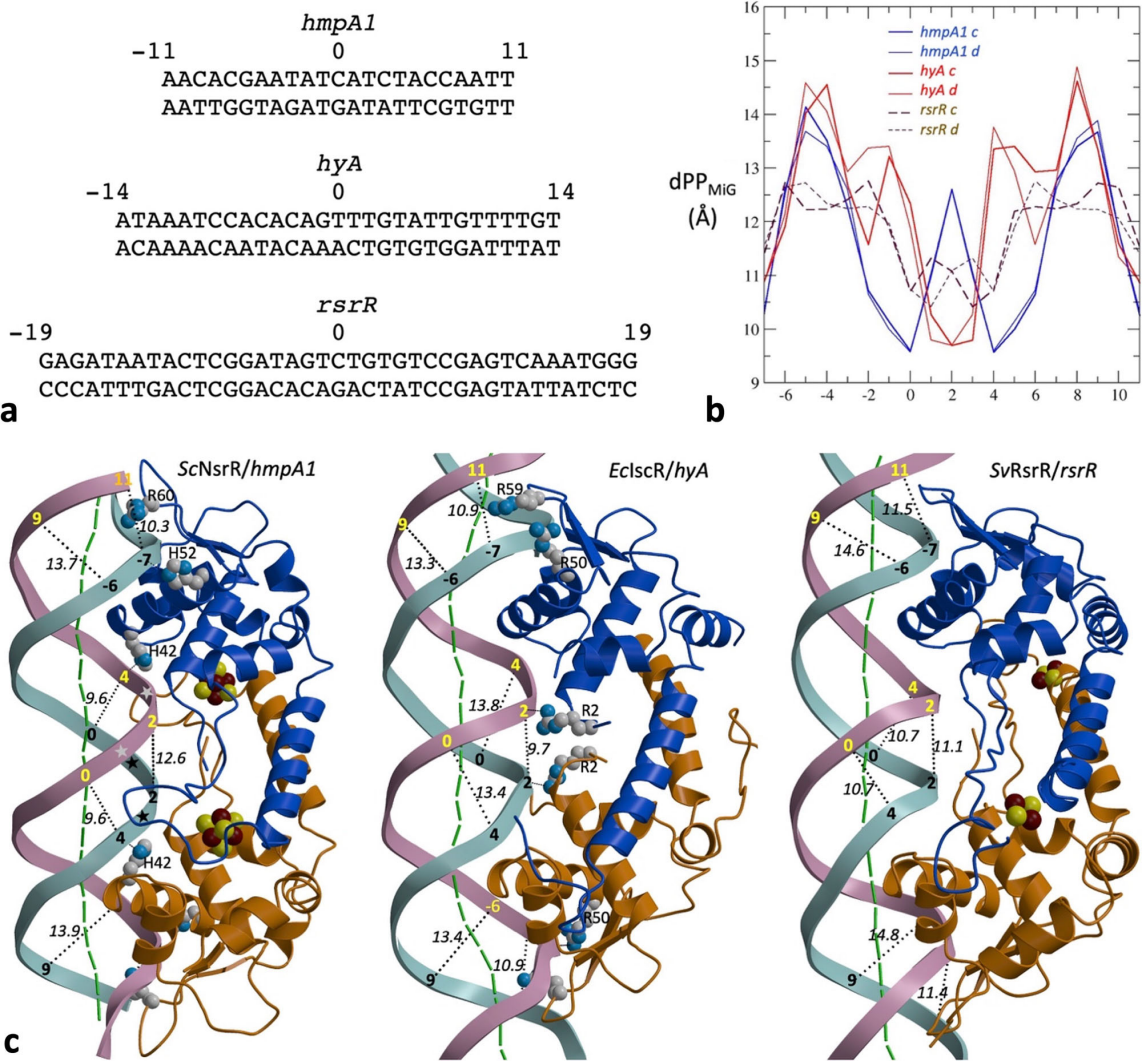
Shape comparison of the DNA complexes of three Rrf2 family members. **a** Base sequence alignment of *hmpA1*, *hyA* and *rsrR* operator fragments in their corresponding complexes with [4Fe-4S]-*Sc*NsrR, apo-*Ec*IscR and [2Fe-2S]-*Sv*RsrR. **b** Comparison of P-to-P distances (dPP_Mig_) in the MiGs of *hmpA1*, *hyA* and *rsrR* for the phosphate groups in both DNA strands. **c** Polypeptide and DNA folding of the three labeled complexes in a view perpendicular to that of Fig. 1. Protein subunits, DNA strands and bp tracing, phosphate positions, cluster atoms and amino acids are represented as in Fig. 1. Black dotted lines trace dPP_Mig_ distances (given in Å). Because of its limited resolution, no protein residues are shown for the *Sv*RsrR-DNA complex.

The DNA double helices of the 39-bp *rsrR*, 29-bp *hyA* and 23-bp *hmpA1* operator fragments display different shapes in their respective complexes to *Sv*RsrR, *Ec*IscR and *Sc*NsrR (Fig. 6). The DNA structures accommodate the different positions and orientations of the H-wHTH motifs found in the bound proteins (Fig. S9). The MiG and MaG P-to-P distances (Fig. 6b, c) and widths (Fig. S10) observed at different locations of the DNA double helix in the three complexes are remarkably different. The MiGs are especially different in the central 7-bp region (Fig. 6b, c) for which no base-specific contacts with the protein are observed (Fig. 2 and Table S1). These groove width variations condition the distance between equivalent operator regions (Fig. S11), which could guide recognition by different Tfs^32,33^.

**Fig. 7:**
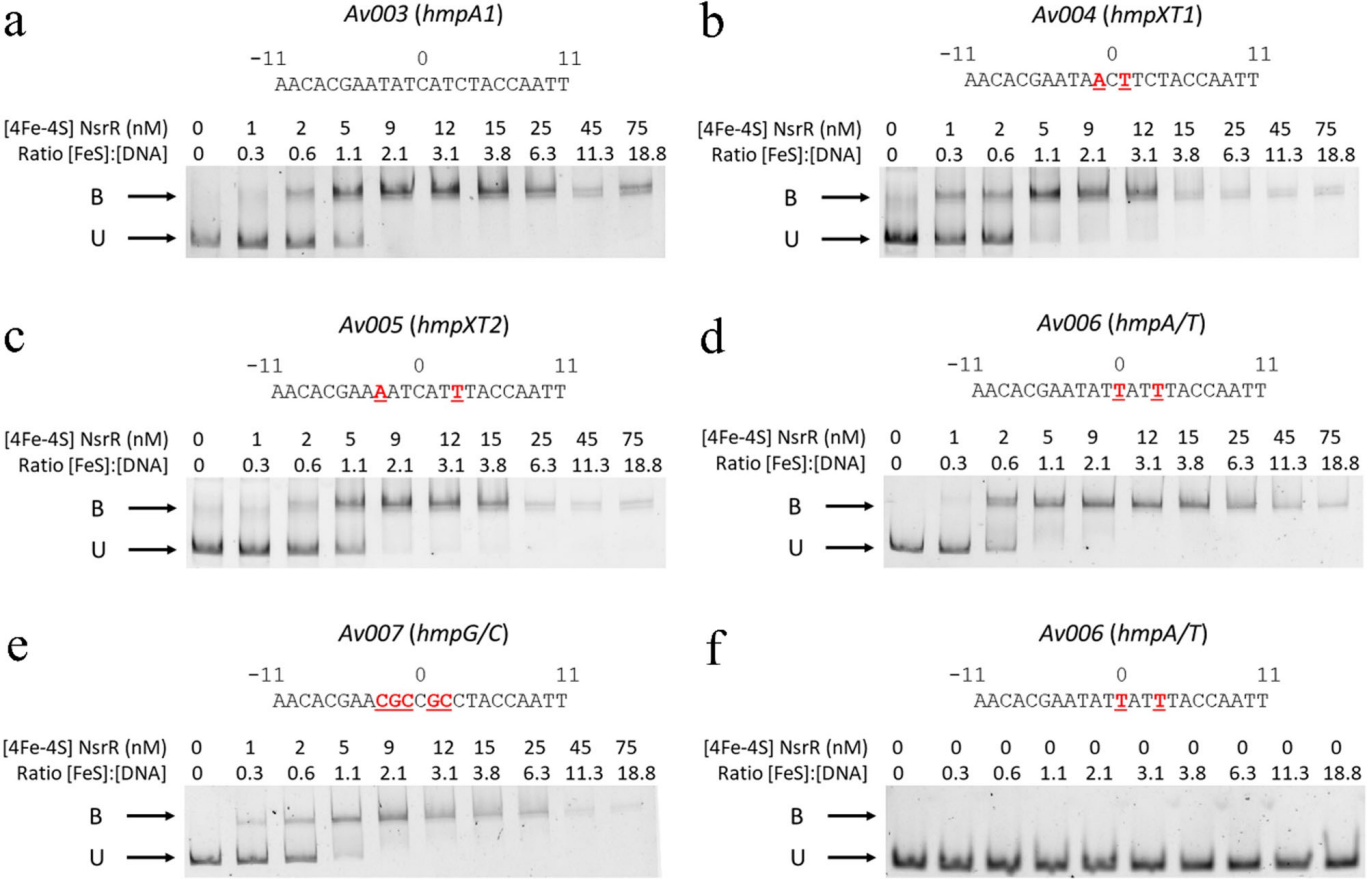
Binding of NsrR to modified probes. DNA binding EMSAs showing probes (Av003 – Av007) unbound (U) or bound (B) by [4Fe-4S] NsrR. Probes contain: **a** Wild type *hmpA1* operator, **b** *hmpXT1*, **c** *hmpXT2*, **d** *hmpA/T*, **e** *hmpG/C* sequences, **f** Protein-free control for *hmpA/T*. Base sequences for each probe are shown, with changes relative to the wild type *hmpA1* site shown in red. The DNA concentration was ~4 nM. Concentration of [4Fe-4S] NsrR used and ratios of [4Fe-4S] NsrR to DNA are indicated. The binding buffer contained 10 mM Tris, 54 mM KCl, 0.3% (v/v) glycerol, 1.32 mM GSH, pH 7.5.

To investigate whether the sequence of the central 7-bp region of *hmpA1* is important for DNA recognition by *Sc*NsrR we performed EMSA binding studies of fragments with modified bases (Fig. 7), embedding the 23-bp consensus central region in 151-bp dsDNA probes (see Methods). Wild type *hmpA1* (Av003) has four dinucleotides in a B_II_ phosphate backbone conformation in the 7-bp central region: C_0c_A_+1c_, T_+2c_C_+3c_, G_0d_A_+1d_ and T_+2d_A_+3d_ (Figs. 1–2 and 6). Because XT dinucleotides have a low probability of being in the B_II_ conformation in regular B-DNA^38^, we replaced A_+1c_ and A_+1d_ by T in probe Av004 and C_+3c_ and A_+3d_ by T in Av005 (see Table S2). The observation that some XT steps are found at the corresponding positions in the aligned *hyA* and *rsrR* operator fragments (Fig. 6a) suggested to us that they could play a discriminating role in DNA recognition between NsrR, IscR and RsrR. However, our EMSA results did not show any clear difference in binding affinity to *Sc*NsrR between the two constructs having XT steps (Av004 & Av005) and the unmodified *hmpA1* (Av003) sequence (Fig. 7a–c). We also checked the binding of variants with an AT-rich (Av006) and a GC-rich (Av007) central region (Fig. 7d–e), because AT-rich base sequences have been associated with an increased likelihood of MiG narrowing^32^, as observed in the central region of the 23-bp *hmpA1* fragment (Figs. 2 and 6). However, again our results showed that *Sc*NsrR binds the modified operators essentially stoichiometrically and we could detect no significant differences in binding affinities under these conditions. It should be noted that variations in the flexibility of the central region caused by A/T or G/C enrichments^39–41^ could affect DNA-protein binding kinetics. However, such an effect would not be detected by the EMSA assay reported here.

## Discussion

In spite of the large buried complex surface, most of the *Sc*NsrR contacts with the *hmpA1* operator are with the phosphate/sugar backbone; only a few direct interactions with nucleobase rings are observed (Figs. 2 and 3). What, then, determines the *hmpA1* specificity for *Sc*NsrR? Like most other DNA-binding proteins, sensors such as NsrR must locate specific targets in order to perform their biological role. Experiments performed with green fluorescent protein (GFP)-tagged *lac* repressor showed it to diffuse along the helical axis of the DNA probe via a sliding mechanism^42^. Furthermore, a similar study using an RNA polymerase demonstrated that this protein tracked the helical pitch along a DNA groove^43^. These interactions, made of non-specific electrostatic contacts of protein residues with the phosphate backbone, are dominated by a fully entropic potential^44^. Indeed, the electrostatic potential at the *Sc*NsrR surface is consistent with such non-specific interactions playing an important role in DNA recognition (Fig. S5). Specific binding will then require a unique DNA sequence and protein-nucleobases interactions conditioned by the *shape* of both partners^45^.

The shape of B-DNA is determined by sequence-dependent variations in minor and major groove width and curvature that are at least partially coupled. Inter bp rolls, defined as rotations around the base pairing axis perpendicular to the double helix axis, are major contributors to DNA curvature^46,47^; zero rolls give straight B-DNA double helices^48^. The *hyA* central 11-bp region is curved, as reflected by an average bp roll of 2.3° (Fig. S1). This value is only 0.5° in *hmpA1*, in which the same region is almost straight. This may be related to the observed presence of four B_II_ backbone conformations in *hmpA1* (Fig. 2). Dinucleotide bps, such as TpC•GpA, with both connecting phosphates in a B_I_ conformation (i.e., B_I_•B_I_) are mostly associated with positive rolls, whereas negative rolls are typically found for B_I_•B_II_ conformations^49^. As a result, the rolls of a sequence of bps with alternating B_I_•B_II_ and B_I_•B_I_ phosphates (like in *hmpA1*) may partially cancel out, producing a smaller average roll than a sequence lacking B_II_ conformations (like in *hyA*). However, our EMSA DNA binding studies (Fig. 7) do not suggest any role, at least in vitro, for the observed B_II_ backbone conformations in *hmpA1* binding to the protein sensor.

Different backbone torsion angle combinations are required for successive dinucleotides to bend in the same direction along the DNA double helix. Factors such as base orientation and stacking, and electrostatic interactions between phosphate groups may influence the torsion angle values. DNA bending towards the MiG, which narrows it, is energetically more costly than its bending towards the MaG^50^. However, the electrostatic repulsion between MiG opposing phosphate groups is greatly reduced by the proximity of positively charged amino acid sidechains. Thus, a minimal MiG width (MiGW) is observed at the + 4 phosphate of *hmpA1* and at the +2 phosphate of *hyA*, which respectively make salt bridges with *Sc*NsrR His42 (assuming it is positively charged) and *Ec*IscR Arg2 (Fig. 6). Narrow MiGs in naked DNA have been associated with A/T bp-rich sequences^32,46^. Stella and coworkers found that the A/T-rich central region of the Fis protein high-affinity DNA binding sites displayed a small MiGW^46^. G/C substitutions in this region increased the MiGW and resulted in lower DNA binding affinity, an effect these authors explained by the increased conformational cost of compressing the MiG. However, B-DNA sequences are known to form dynamically heterogenous structures that explore a large conformational space^51^. When we performed an EMSA binding study of a variant of *hmpA1* with 7 G/C bps in the central region, we did not observe a change in binding affinity to *Sc*NsrR (Fig. 7). Neither did we see an effect when the region was enriched in A/T bps. This may imply that the flexibility of the *hmpA1* DNA central region of the structure does not have a marked effect on the thermodynamics of the recognition of the operator -through either induced fit or conformational selection- by the different shapes and electrostatic potentials at the *Sc*NsrR surface. A possibly kinetic effect would not be detected in the EMSA.

In view of these results, *direct* protein-to-DNA interactions, which mostly involve the RH and the wing (Figs. 2 and 6), are probably much more important for operator recognition. The DNA segments bending around the regulatory helix display a widening of the MaG. This widening is more pronounced in *hyA* than in *hmpA1* and *rsrR* (Fig. S10), which could be due to the compression of the MiG between the P_+2_ phosphates of the neighboring central region in *hyA* (Fig. 6). The wing regions of *Sc*NsrR, *Ec*IscR and *Sv*RsrR, which display the highest amino acid sequence homologies within Rrf2 family proteins (Fig. 5), interact with a more similar, relatively A/T-rich MiG (Figs. 2 and 6a). In *hmpA1*, as in *hyA*, the corresponding MiGWs are narrower than in *rsrR*. This is probably due to the close interaction of the positively charged *Sc*NsrR Arg60, and *Ec*IscR Arg59, with deoxyriboses and base rings at the MiG edge (Fig. 6c and Table S1). These interactions also compensate for the electrostatic repulsion between opposing phosphate groups. The broader *rsrR* MiG in the region that binds the protein wing could be explained by its lower A/T bp content and by the fact that in *Sv*RsrR this Arg residue is substituted by Gln57 (Fig. 5).

To further analyze DNA binding specificity, we modeled the *hyA* and *rsrR* operator sequences (Fig. 6a) on that of *hmpA1* in its complex with *Sc*NsrR. Substituting *hmpA1* C_+6_ by (*hyA*) T_+6_ introduces a vdW (*d* = 2.7 Å) collision between the added C7 methyl group and the Cγ2 methyl of (*Sc*NsrR) Thr41 (Table 2). Because the latter is located in the N-terminal region of the RH and its Oγ1 atom makes a H-bond with the 5^’^-phosphate group of the same base in *hmpA1* (Table S1), other interactions involving the RH are likely to be perturbed as well. The same substitution in the other DNA strand should similarly modify the RH of the corresponding *Sc*NsrR subunit, which could be sufficient to abolish binding. Similarly, when modeled on the structure of *hyA*, the C7 methyl group of T_+5_ found in one strand of *hmpA1* and in both strands of *rsrR* lies within 2 Å from the side chain of Ser40 of *Ec*IscR (Table 2). In addition, the T_-8d_ C7 methyl group of *hmpA1* would collide with the carboxylate group of *Ec*IscR Glu43, which has been assigned an important role in DNA recognition^17^. These collisions suggest that *hmpA1* and *rsrR* are not recognized by *Ec*IscR. Because of its relatively low resolution^21^, we refrained from doing a similar analysis using the structure of the *Sv*RsrR/*rsrR* complex.

The observed interaction of Pro61 in the wing of *Ec*IscR with the MiG of a 29 bp fragment of *hyA* (Table S1) suggests that the aligned Arg62 of *Sc*NsrR could bind to the MiG of a longer than 23 bp fragment of *hmpA1*, as the one used in our EMSA studies which is 151 bp-long (Fig. 7). This should increase binding affinity. Substitution of (*hmpA1*) T_+10_ by (*rsrR*) G_+10_ introduces vdW collisions with (*Sc*NsrR) Arg60 in both wing regions (Table 2). We also noted that the last three Gly residues of the conserved Lx_3_Gx_6_Gx_2_GGx_2_L wing motif directly interact with the DNA in the *Ec*IscR/*hyA* complex structure (Table S1). This further underscores the importance of the wing region for DNA binding.

There are few structural changes in [4Fe-4S]-*Sc*NsrR upon its binding to the *hmpA1* operator (Fig. 4a). As mentioned in the Introduction, the *nsrR* gene codes for *Sc*NsrR, whereas the two *hmpA* genes encode NO-detoxifying flavohemoglobins. Full binding of the [4Fe-4S]-*Sc*NsrR dimer to 23 bp DNA stretches -included near the center of 267 bp constructs- as investigated by earlier reported EMSA studies^25^, requires corresponding ratios of 1:1 for *hmpA1*, 2.5:1 for *nsrR* and 4:1 for *hmpA2*. NO-induced structural changes resulting from iron-sulfur cluster modification and degradation will alter the *Sc*NsrR structure and, consequently, modify its binding affinity. The [4Fe-4S]-*Sc*NsrR TF binds to its known operators^25^ with much higher affinity than either non-physiological [2Fe-2S]-*Sc*NsrR species^29^ or its apo-form. We have provided structural data to show that the loss of the iron-sulfur cluster in the latter introduces radical structural differences relative to the holo protein form at the DNA-interacting region^22^. Large conformational changes, including the possible formation of persulfide bonds^27^, should also be present in [2Fe-2S]-*Sc*NsrR; indeed these bonds have been observed in partially oxidized [2Fe-2S] clusters of HydE^52^ and detected by resonance Raman spectroscopy in FNR^53^.

We have found that the DNA-free [4Fe-4S]-*Sc*NsrR structure does not change much after binding of the 23 bp *hmpA1* operator fragment and that changing the sequence of its central 7-bp region (which shows no direct base-protein contacts within 4 Å) has no effect on the binding affinity. Consequently, it seems reasonable to assume that the DNA backbone conformation of bound *hmpA2* and *nsrR* operators is not very different from that of *hmpA1*. We have therefore modeled the *hmpA2, nsrR* and (*Ec*) *hmpA* sequences on that of *hmpA1*, knowing that (*Ec*) *hmpA* binds only very weakly to *Sc*NsrR^25^. Analysis of the resulting predicted complex structures shows a number of bad contacts involving Arg60 in the two NsrR wing regions. Together, pointing into the minor groove at the two extremes of the *hmpA1* operator fragment, Arg60_A_ and Arg60_B_ make close (<4 Å) contacts with eleven nucleotides (Fig. 2 and Table S1). A collision with one of these nucleotides should perturb the interactions with the other ones. Three bad (d < 2.85 Å) vdW contacts with Arg60 are obtained for the modeled *Sc*NsrR/*Ec-hmpA* complex (Tables S3–S4 and Fig. S12), which could explain why no significant binding is observed for this operator. One bad contact with the Arg60 guanidinium group is obtained for *nsrR*, in line with its reduced binding affinity. The observed low affinity of *hmpA2* is possibly explained by the substitution of T_-9d_ by C and T_+5d_ by G causing a loss of favorable vdW contacts with the T-C7 methyl groups.

Holo-*Sc*NsrR-operator binding is abolished for NO-to-[4Fe-4S]-cluster ratios of about 2 for *hmpA2*, 4 for *hmpA1* and 8 for *nsrR*^24^. As in the case of O_2_ oxidation, important conformational changes at the cluster region are expected when intermediate NO-bound degradation products are formed. Kinetic and spectroscopic studies have shown that these intermediates appear at ratios of about 2, 4, and 6 bound NOs per cluster^24^. They share spectral features with known NO-iron complexes such as [Fe(NO)_2_(RS)_2_] (dinitrosyl iron complex), [Fe_2_(NO)_4_(RS)_2_] (Roussin’s red ester) and [Fe_4_(NO)_7_(S)_3_] (Roussin’s black salt, RBS)^24,26^. In the case of the RBS-like intermediate, it has been suggested that Cys thiolates (or persulfide derivatives of these) replace sulfide ligands^26,28^; this would explain how these nitrosylated moieties bind to the protein after the loss of cluster sulfide ions. Several NO-modified cluster forms have also been characterized by mass spectrometry, including early mono- and di-nitrosyl intermediates of the [4Fe-4S] cluster^28^. At higher NO-to-cluster ratios, persulfide adducts accumulate, with a gradual breakdown of the [4Fe-4S] core to form [Fe_2_(NO)_4_] and [Fe_2_(NO)_4_S] adducts^27^. The *Sc*NsrR-*hmpA1* complex starts to dissociate at ~2 NO per [4Fe-4S] cluster, where only early intermediates of nitrosylation are observed. This suggests that relatively minor modifications of the cluster can abolish operator binding. The comparison of both non-complexed and complexed [4Fe-4S]-*Sc*NsrR to apo-*Sc*NsrR^22^ has defined the nature of the conformational changes induced by either iron-sulfur cluster modification or loss that will preclude sensor binding to *hmpA1* (Fig. 4b). Because we have not yet characterized any NO-complex of *Sc*NsrR, we have at this stage no structural data to explain the different NO sensitivities of its complexes with *hmpA2*, *hmpA1* and *nsrR*.

In conclusion, the main determinant factors for the *Sc*NsrR base sequence specificity can be described as follows: (i) the end regions of the *hmpA1* operator interact extensively with the *Sc*NsrR wings (this is also the case for *hyA* and *rsrR*, which respectively bind the apo-*Ec*IsrR and [2Fe-2S]-*Sv*RsrR wings); (ii) important structural differences between the bound operators of these three Rrf2 family members reside in variations of minor and major groove widths, which result in different phosphate positions; (iii) the DNA also displays specific curvatures around the RH of the sensor that properly orient its phosphate groups to form salt bridges and hydrogen bonds with equivalent side chains from both protein subunits; (iv) much of the specificity involves a relatively small number of direct interactions of protein side chains with nucleobase rings; (v) as shown in the EMSA experiments, changes in the flexibility of the *hmpA1* central region do not affect the thermodynamics of NsrR binding (whether they modify the kinetics of this process was not investigated here). Solvent-mediated interactions may also play a role, but these are not resolved in our 3 Å resolution X-ray model. Our results extend the findings of a recently reported sequence similarity network analysis of the entire Rrf2 superfamily that places *Sc*NsrR, *Ec*NsrR, *Ec*IscR and *Sv*RsrR in distinct cluster configurations^54^. Our next challenges will be to crystallize complexes of [4Fe-4S]-*Sc*NsrR with longer constructs of the *hmpA1*, *hmpA2* and *nsrR* operators and solve their structures at the highest possible resolution. These structures should help us determine if and how the above-described determinants vary with the nature of the operator. In addition, we aim now at structurally characterizing NO-induced changes for at least one *Sc*NsrR/DNA complex.

## Methods

### Crystallization

[4Fe-4S]-*Sc*NsrR was purified under strictly anaerobic conditions as described previously^22,25^. A stock solution with a protein concentration of 43 mg/mL in 50 mM Tris pH 8.0, 2 M NaCl, 5% glycerol was used for all experiments. A 23 bp dsDNA fragment of the *hmpA1* operator was prepared by annealing its 5^’^-AACACGAATATCATCTACCAATT-3^’^ and 5^’^-AATTGGTAGATGATATTCGTGTT-3^’^ complementary oligonucleotides in DNase-free water (Alfa Aesar), as already described previously^24^. [4Fe-4S]-*Sc*NsrR was complexed with the 23 bp *hmpA1* operator by incubating a solution of 17 mg/mL of the protein mixed with a 3-fold excess of the dsDNA fragment in 50 mM Tris pH 8.0, 500 mM NaCl, 5% glycerol for 60 minutes at 20 °C. Crystals were obtained by screening 864 different conditions from commercial kits in 96-well plates with a Gryphon robot (Art Robbins Instruments, CA, USA) installed inside an anaerobic glove box, using the vapor diffusion method. For each crystallization condition a drop was set by the robot by mixing 200 nL of the solution of the [4Fe-4S]-*Sc*NsrR/*hmpA1* complex with 200 nL of the commercial reservoir solution. The sitting drop was then equilibrated against 100 μL of the latter solution. Manual optimization of the best automated crystallization condition yielded usable crystals from hanging drops prepared by mixing 1 μL of the protein-DNA mix with 1 μL of the reservoir solution containing 27% PEG 550 MME, 13.5% PEG 20000, 30 mM MgCl_2_, 30 mM CaCl_2_ and 100 mM MOPS/HEPES pH 8.0 equilibrated against 1 mL of this solution. All these experiments were performed anaerobically in a glove box.

### X-ray data collection

Crystals were transferred from the original drop to the crystallization solution, with or without 20% v/v glycerol added as cryoprotectant. They were then fished with a cryo-loop, flash-cooled in liquid propane inside the glove box^55^ and transferred outside for storage in liquid nitrogen. Initial X-ray diffraction data were collected to 4.1 Å resolution for a crystal mounted from a cryo-protected solution, while maintaining it under a N_2_ stream at 100 K at beamline PX-2A of the SOLEIL synchrotron (Saint-Aubain, France). Data to about 3.0 Å resolution were subsequently obtained from crystals flash-cooled without added glycerol. The best data set (Table 1) was collected at a temperature of 100 K and a wavelength of 1.00001 Å with an Eiger X 9 M (Dectris) detector at beamline X06SA of the Swiss Light Source (Villigen, Switzerland), using the DA+ software^56^. The diffraction data were indexed, integrated and scaled with the XDS package^57^ and the program AIMLESS^58^ was used to check for anisotropic diffraction. Although X-ray data reduction initially indicated that the crystal belonged to the hexagonal P6_5_22 space group, further analysis showed it to be P6_5_. The change in Laue class from 6/mmm to 6/m of the diffraction data set results from the fact that the dsDNA fragment is not a perfect palindrome; consequently, it does not display the twofold symmetry of the protein dimer. The intensity statistics of the data showed no evidence of twinning.

### Structure determination

The structure of the [4Fe-4S]-*Sc*NsrR/*hmpA1* complex was solved by molecular replacement (MR) with PHASER^59^ using two search models: the known non-complexed protein structure^22^ and the 23 bp *hmpA1* operator. The latter was constructed with B-DNA geometry using COOT^60^. The obtained MR solutions were confirmed by high peaks in F_obs_-F_calc_ and anomalous difference electron density maps for the *Sc*NsrR [4Fe-4S] clusters, which were not included in the protein search model. The resulting model was subjected to rigid body, positional, TLS and B-factor refinement with the PHENIX and Refmac5 programs^61,62^, using COOT^60^ for manual corrections. Non-crystallographic symmetry and secondary structure restraints were used for positional refinement. H-bond restraints were included for DNA base pairing interactions and some protein-DNA contacts. The refinement procedure gave best results when defining the *hmpA1* 23 bp operator and two protein half-dimers as TLS groups. No hydrogens were included in the model. Each half-dimer consisted of NsrR residues 1–86 and 124–144 from one subunit and residues 87–123 from the other, including also the [4Fe-4S] cluster bound between them. The final model has good refinement statistics, stereochemistry (Table 1) and a low all-atom clash score of 3.8, as measured by MolProbity^63^. There are 0 outliers in the Ramachandran (φ-ψ) plot and 2.3% rotamer outliers. The dsDNA fragment fits well to the electron density map but a relatively poor match is observed for about 30% of the protein model due to disorder, as indicated by high B-factors. However, all *Sc*NsrR side chains forming hydrogen bonds or salt bridges with the dsDNA have good matching electron density. We tried to explain residual peaks found at both ends of the *hmpA1* operator fragment in the final F_obs_-F_calc_ map by adding additional ordered nucleotides to the model. However, this resulted in an increase of the R_free_. Because a threefold excess of DNA was used for crystallization, we tentatively assign these residual peaks to partially disordered non-complexed DNA. Other operator sequences were modeled with COOT^60^ on the available structures of *hmpA1* (this study) and *hyA*^17^ without changing the structure of the DNA backbone.

### Structural analyses

Buried surface areas (BSAs) between the various protein and DNA chains were determined with the program Pisa^64^. DNA geometry, including the measurement of torsion angles, bp origins, rolls and propeller twists, was analyzed with DSSR^37^, with groove widths calculated from phosphate-P positions as defined by El Hassan & Calladine^65^ (Fig. S10). We also used the in-house program CavEnv (incorporated in the CCP4 package^66^), which takes into account all atoms of the structure with an atom-type dependent vdW radius, to investigate cavities and groove widths (Fig. S7). Figures were prepared with either COOT^60^, Bobscript^67^ and Raster3D^68^, PyMOL (www.pymol.org, the PyMOL molecular graphics system, version 2.1 Schrödinger, LLC) or manually. Compilations of all the used programs were provided by SBGrid^69^. The A/T notation for bps and AT for base steps is used throughout, with a number and one letter for the strand added as an index in some cases. Nucleotides are numbered with respect to the central bp, which is assigned the value zero. Amino acid residues are shown using three-letter codes in the text and one-letter codes in the figures and tables. Structures of the *Ec*IscR/*hyA* and *Sv*RsrR/*rsrR* complexes were obtained from deposition codes 4HF1 and 6Y42 in the Protein Data Bank^70^.

### EMSA (electrophoretic mobility shift assay) binding studies

DNA fragments carrying wild type or mutated *hmpA1* promoter sequences (Table S2) were PCR-amplified using a 151-base-pair double-stranded DNA template, 6-carboxyfluorescein (6-FAM) modified primers (Metabion, Germany) and Phusion Hot Start II DNA Polymerase (Thermo Scientific). The PCR products were purified using NucleoSpin Gel and PCR Clean‑up kit (Macherey-Nagel) according to the manufacturer^’^s instructions. The concentration of the 6-FAM labeled probes was determined using a Varian Cary 50 UV-Vis spectrophotometer. The molecular weights and 260 nm absorption coefficients for 6-FAM labelled probes were calculated using OligoCalc^71^. EMSA reactions (20 μl) were carried out on ice in 10 mM Tris, 54 mM KCl, 0.3% (v/v) glycerol, 1.32 mM glutathione, pH 7.5, as previously described^25^. Briefly, 2 μl of each DNA probe was titrated with varying aliquots of [4Fe-4S] NsrR, to a ~20-fold molar excess. Loading dye (2 μl of 0.3% (w/v) bromophenol blue, 50% (w/v) glycerol) was added and the reaction mixtures were immediately separated at 30 mA for 30 min on a 5% (w/v) polyacrylamide gel in 1 × TBE (89 mM Tris, 89 mM boric acid, 2 mM EDTA). Gels were visualized on a Typhoon FLA9000 (GE Healthcare). Polyacrylamide gels were pre-run at 30 mA for 2 min prior to use. Each probe has a dimeric NsrR binding site with one [4Fe-4S] cluster per protein subunit. As apo-NsrR does not bind DNA^25^, concentrations are presented as [4Fe-4S] per DNA, as indicated in Fig. 7.

### Statistics and reproducibility

X-ray diffraction data collection and refinement statistics for the reported NsrR/*hmpA1* complex are given in Table 1. EMSAs for each tested DNA fragment were carried out at least twice, on different days, to ensure repeatable observations. The images shown in Fig. 7 are the best representative images for each EMSA probe.

### Reporting summary

Further information on research design is available in the Nature Research Reporting Summary linked to this article.

## Supplementary information

Supplementary Information

Reporting Summary

## Data Availability

Atomic coordinates and structure factors for the reported crystal structure have been deposited with the Protein Data Bank^70^ (https://www.rcsb.org) under accession number 7B0C. A structure validation report is also available there. The two other structures used in our paper are available in the Protein Data Bank (pdb) under accession codes 4HF1 and 6Y42, as given in Fig. 5. These pdb depositions were used to generate all the figures given in the main manuscript, as stated above in the section Structural Analyses, except for the manually prepared Fig. 5 and the measured EMSAs in Fig. 7. For the latter, uncropped images are included in the Supplementary Material as Fig. S13. Interatomic distances in Figs. 1–3, 6b, and c were measured from the atomic coordinates in the pdb depositions given above. Amino acid sequences in Fig. 5 were obtained from the pdb deposition codes mentioned in its last colum or from the UniProt database (https://www.uniprot.org, codes Q1R382 for *Ec*NsrR and Q1ML82 for *Rl*RirA).
