# Supplementary material for: Structural determinants of DNA recognition by the NO sensor NsrR and related Rrf2-type [FeS]-transcription factors
Source: Commun Biol. 2022 Jul 30;5:769. doi: 10.1038/s42003-022-03745-7 (PMC9338935; doi:10.1038/s42003-022-03745-7)
Supplement: Supplementary file 1 — Supplementary Information [file 42003_2022_3745_MOESM1_ESM.pdf]

SUPPLEMENTARY MATERIAL

Structural determinants of DNA recognition by the NO sensor NsrR and related Rrf2-type [FeS]-transcription factors

Roman Rohac, Jason C. Crack, Eve de Rosny, Océane Gigarel, Nick E. Le Brun, Juan C. Fontecilla-Camps, Anne Volbeda

Table S1. Comparison of [4Fe-4S]-*Sc*NsrR/*hmpA1* and apo-*Ec*IscR/*hyA* < 4.0 Å contacts.*

base nr	***hmpA1*$_C$**	***Sc*NsrR**	dist (Å)	***hmpA1*$_D$**	***Sc*NsrR**	dist (Å)	***hyA*$_C$**	***Ec*IsrR**	dist (Å)	***hyA*$_D$**	***Ec*IsrR**	dist (Å)
-11							A-N3	$P_{61B}C\delta$	3.6	A-N3	$P_{61A}C\delta$	3.5
-10	A-N3	$R_{60A}O$	3.0	A-N3	$R_{60B}O$	3.0	A-O5'	$P_{27B}C\beta$	3.6	A-O5'	$P_{27A}C\beta$	3.8
-10	A-C1'	$R_{60A}O$	3.2	A-C1'	$R_{60B}O$	3.2				A-O4'	$G_{60A}O$	3.5
-10										A-C5'	$G_{63A}O$	3.7
-10										A-O3'	$G_{64A}N$	3.7
-9	C-OP1	$T_{29A}N$	**3.1**	T-OP1	$T_{29B}N$	**3.1**	T-OP1	$L_{28B}N$	**2.8**	A-OP1	$L_{28A}N$	**2.9**
-9	C-OP1	$T_{29A}O\gamma1$	**2.8**	T-OP1	$T_{29B}O\gamma1$	**2.9**	T-OP1	$L_{28B}C\gamma$	3.5	A-OP1	$L_{28A}C\gamma$	3.7
-9	C-OP1	$L_{66A}C\gamma$	3.7	T-OP1	$L_{66B}C\gamma$	3.8	T-OP1	$Y_{65B}C\varepsilon2$	3.5	A-OP1	$Y_{65A}C\varepsilon1$	3.4
-9	C-C5'	$V_{59A}O$	3.3	T-C5'	$V_{59B}O$	3.3						
-9	C-O3'	$V_{58A}C\beta$	3.5	T-O3'	$V_{58B}C\beta$	3.5	T-O3'	$S_{57B}C\beta$	3.7	A-O3'	$S_{57A}C\beta$	3.6
-9	C-O4'	$R_{60A}C\gamma$	3.4	T-O4'	$R_{60B}C\gamma$	3.4	T-OP1	$G_{64B}C\alpha$	3.6	A-OP1	$G_{64A}C\alpha$	3.3
-9	C-O2	$R_{60A}N\eta2$	**2.8**	T-O2	$R_{60B}N\eta2$	**2.8**	T-O2	$R_{59B}N\eta2$	**3.3**	A-N3	$R_{59A}N\eta2$	3.4
-9				T-C7	$Y_{40B}O\eta$	3.7						
-8	A-OP1	$V_{58A}C\beta$	3.7	T-OP1	$V_{58B}C\beta$	3.7	C-OP1	$S_{57B}O\gamma$	**2.7**	C-OP1	$S_{57A}O\gamma$	**2.6**
-8	A-OP1	$Q_{51A}C\gamma$	3.2	T-OP1	$Q_{51B}C\gamma$	3.2	C-OP1	$R_{50B}N\varepsilon$	3.4	C-OP1	$R_{50A}N\varepsilon$	**2.9**
-8	A-OP2	$T_{48A}O\gamma1$	**3.0**	T-OP2	$T_{48B}O\gamma1$	**3.0**						
-8	A-OP2	$T_{47A}C\gamma2$	3.7	T-OP2	$T_{47B}C\gamma2$	3.6	C-OP2	$Y_{65B}O\eta$	**2.7**	C-OP2	$Y_{65A}O\eta$	**2.6**
-8	A-O4'	$R_{60A}N\eta2$	**2.9**	T-O4'	$R_{60B}N\eta2$	**3.0**	C-C5'	$R_{59B}N\eta1$	3.5	C-C5'	$R_{59A}N\eta1$	3.5
-8							C-N4	$E_{43B}O\varepsilon2$	**2.7**	C-N4	$E_{43A}O\varepsilon2$	**2.8**
-7	C-OP2	$H_{52A}N\varepsilon2$	**2.6**	G-OP2	$H_{52B}N\varepsilon2$	**2.6**	C-N4	$E_{43B}O\varepsilon1$	**3.1**	A-N6	$E_{43A}O\varepsilon1$	3.5
-7	C-OP2	$T_{48A}C\gamma2$	3.3	G-OP2	$T_{48B}C\gamma2$	3.2						
-6	G-N7	$K_{45A}N\zeta$	3.2	G-N7	$K_{45B}N\zeta$	3.2						
-6	G-O6	$K_{45A}N\zeta$	3.5	G-O6	$K_{45B}N\zeta$	3.3						
-5							C-N4	$Q_{44B}N\varepsilon2$	3.9			
1							C-O3'	$R_{2A}N\eta2$	3.9			
2							T-OP1	$R_{2A}N\varepsilon$	**3.1**	T-OP1	$R_{2B}N\eta2$	**3.2**
2	T-C5'	$T_{4B}C\gamma2$	3.9	T-C5'	$T_{4A}C\gamma2$	4.0	T-OP1	$T_{4A}O\gamma1$	3.5	T-OP1	$T_{4B}O\gamma1$	3.5
2							T-OP2	$R_{2A}N\eta2$	3.1			
2							T-OP2	$K_{6A}C\delta$	3.5			
3	C-OP1	$K5_{B}N$	**2.7**	A-OP1	$K5_{A}N$	**2.8**	G-OP1	$S_{5A}O\gamma$	**2.5**	G-OP1	$S_{5B}C\beta$	3.3
3	C-OP2	$F6_{B}N$	**3.0**	A-OP2	$F6_{A}N$	**2.9**	G-OP2	$K_{6A}N$	3.5	G-OP2	$K_{6B}N$	3.6
3							G-OP1	$Y_{9A}C\varepsilon2$	3.3	G-OP1	$Y_{9B}C\varepsilon2$	3.9
3	C-OP2	$H_{42B}C\varepsilon1$	3.6	A-OP2	$H_{42A}C\varepsilon1$	3.5	G-OP2	$Y_{41A}O\eta$	**2.6**	G-OP2	$Y41_{B}O\eta$	**2.7**
4							T-OP2	$S_{38A}N$	**3.2**	T-OP2	$S_{38B}N$	3.3
4	T-OP1	$P_{39B}C\gamma$	3.7	T-OP1	$P_{39A}C\gamma$	3.4	T-OP2	$S_{38A}O\gamma$	**2.8**	T-OP2	$S_{38B}O\gamma$	**2.5**
4	T-OP2	$H_{42B}N\delta1$	**2.6**	T-OP2	$H_{42A}N\delta1$	**2.7**						
4							T-O4	$Q_{44A}N\varepsilon2$	**3.2**	T-O4	$Q_{44B}N\varepsilon2$	**3.0**
4	T-C7	$H_{42B}C\delta2$	3.5	T-C7	$H_{42A}C\delta2$	3.6	T-C7	$Y_{41A}C\alpha$	3.7	T-C7	$Y_{41B}C\alpha$	3.7
5	A-OP2	$P_{39B}C\beta$	3.3	T-OP2	$P_{39A}C\beta$	3.3						
5	A-OP2	$T_{41B}O\gamma1$	**2.6**	T-OP2	$T_{41A}O\gamma1$	**2.5**	A-N7	$S_{40A}O\gamma$	**2.9**	G-N7	$S_{40B}O\gamma$	**2.7**
5	A-C8	$T_{41B}O\gamma1$	3.6	T-C7	$T_{41A}C\beta$	3.6	A-N6	$S_{40A}O\gamma$	3.5			
9				G-N2	$R_{60A}N\eta2$	3.2						
10	T-O2	$R_{60B}C\delta$	3.4	T-O2	$R_{60A}C\delta$	3.3	T-O2	$R_{59A}N\eta2$	**3.3**	T-O2	$R_{59B}N\eta2$	3.5
11	T-O2	$G_{61B}C\alpha$	3.6				T-O2	$G_{60A}C\alpha$	3.5	T-O2	$G_{60B}C\alpha$	3.4
11	T-O4'	$R_{60B}C\delta$	3.2	T-O4'	$R_{60A}C\delta$	3.2	T-O4'	$R_{59A}N\varepsilon$	3.5	T-O4'	$R_{59B}N\varepsilon$	3.5
12							T-O4'	$R_{59A}O$	3.6	T-C4'	$R_{59B}O$	3.8
12							T-O4'	$G_{60A}C\alpha$	3.6	T-O4'	$G_{60B}C\alpha$	3.5
12							T-O2	$P_{61A}C\delta$	3.1	T-O2	$P_{61B}C\delta$	3.1
13							G-C4'	$P_{61A}O$	3.7	A-O4'	$P_{61B}C\beta$	3.6
							G-N3	$P_{61A}C\gamma$	3.9			

[*] Contact distances ≤ 3.3 Å for H-bonds and salt bridges are given in bold; base and pyrimidine/purine specific atoms are highlighted in green and yellow, respectively. Similar protein contacts to the *hmpA1* and *hyA* operators are highlighted in gray.

Table S2. List of PCR primers and templates used in this study.*

Name	Description	Sequence (5'→3')
AV001	Fwd unversial 6-FAM primer for PCR	CTAAAACGACGGCCAGT
AV002	Rev universal 6-FAM primer for PCR	CAGGAAACAGCTATGAC
AV003	hmpA1 template for PCR	CTAAAACGACGGCCAGTCGGGATGACGGGCGGCGGCCCCCTGAGGCCGTC GAGCTGTGGCCTAA**AACACGAATATCATCTACCAATT**AAGGAGTCGCTGT GCTCTCCGAACAGTCCGTTCCCGTGGTCCGAGCCGTCATAGCTGTTTCCTG
AV004	hmpXT1 template for PCR	CTAAAACGACGGCCAGTCGGGATGACGGGCGGCGGCCCCCTGAGGCCGTC GAGCTGTGGCCTAA**AACACGAATAACTTCTACCAATT**AAGGAGTCGCTGT GCTCTCCGAACAGTCCGTTCCCGTGGTCCGAGCCGTCATAGCTGTTTCCTG
AV005	hmpXT2 template for PCR	CTAAAACGACGGCCAGTCGGGATGACGGGCGGCGGCCCCCTGAGGCCGTC GAGCTGTGGCCTAA**AACACGAAAATCATTTACCAATT**AAGGAGTCGCTGT GCTCTCCGAACAGTCCGTTCCCGTGGTCCGAGCCGTCATAGCTGTTTCCTG
AV006	hmpA/T template for PCR	CTAAAACGACGGCCAGTCGGGATGACGGGCGGCGGCCCCCTGAGGCCGTC GAGCTGTGGCCTAA**AACACGAATATTATTTACCAATT**AAGGAGTCGCTGT GCTCTCCGAACAGTCCGTTCCCGTGGTCCGAGCCGTCATAGCTGTTTCCTG
Av007	hmpG/C template for PCR	CTAAAACGACGGCCAGTCGGGATGACGGGCGGCGGCCCCCTGAGGCCGTC GAGCTGTGGCCTAA**AACACGAACGCCGCCTACCAATT**AAGGAGTCGCTGT GCTCTCCGAACAGTCCGTTCCCGTGGTCCGAGCCGTCATAGCTGTTTCCTG

* Universal 6-FAM labelled primers (AV001-AV002) were used to produce 6-FAM labelled EMSA probes from DNA templates (AV003-AV007). Central NsrR binding site (bold, underlined) and introduced modifications (red) are indicated.

Table S3. *Sc*NsrR *hmpA1* base specificity versus *hmpA2*, *nsrR* and (*Ec*) *hmpA*.*

base	***hmpA1***	d (Å)	***Sc*NsrR**	*nsrR*	d (Å)	*hmpA2*	d (Å)	*hmpA* (*Ec*)	d (Å)
-10d	A-N3	3.0	$R_{60B}O$					*T-O2*	*2.8*
**-9c**	C-O2	2.8	$R_{60A}N\eta 2$	*G-N2*	*2.6*			*G-N2*	*2.6*
**-9d**	T-O2	2.8	$R_{60B}N\eta 2$			C-O2	2.8	*G-N2*	*2.5*
-9d	T-C7	3.7	$Y_{40B}O\eta$			C-C5	4.7	G-N7	5.1
-7c	C-C5	5.1	$T_{48A}O\gamma 1$			A-N7	5.6	T-C7	3.7
-7d	G-N7	5.4	$T_{48B}O\gamma 1$	C-C5	4.9			T-C7	3.5
+5c	A-N7	3.9	$T_{41B}O\gamma 1$	G-N7	3.9	C-C5	3.5		
+5d	T-C7	3.6	$T_{41A}C\beta$			G-C8	4.1	G-C8	4.1
+9c	A-C2	4.2	$R_{60B}N\eta 2$			G-N2	2.8	C-O2	4.9
+9d	G-N2	3.2	$R_{60A}N\eta 2$	C-O2	5.4			C-O2	5.4
+10c	T-O2	3.4	$R_{60B}C\delta$					A-N3	3.4
+11c	T-O2	3.6	$R_{60B}O$					A-N3	3.6

* The latter are modeled on the known structure of the *hmpA1*/*Sc*NsrR complex. For repulsive vdW interactions the contact distance (d) and corresponding base substitution are in *red*. No distance is given when the base is the same as in *hmpA1*. Distances larger than 4 Å are shown in gray. We do not assign the 2.8 Å distance between *hmpA2* $G_{+9c}N2$ and $R_{60B}N\eta 2$ as a bad contact because the other DNA strand of *hmpA1* has also a G at this position.

Table S4. Sequence alignment of (*Sc*)[1] *hmpA1*, *nsrR*, *hmpA2* and (*Ec*)[2] *hmpA*.*

	-11 0 11
hmpA1	AACACGAATATCATCTACCAATT
	TTGTGCTTATAGTAGATGGTTAA
nsrR	AAGGCGAACCTAGCATGCGCATT
	TTCCGCTTGGATCGTACGCGTAA
hmpA2	AACAAGCATCTGAGATCCCAGTT
	TTGTTCGTAGACTCTAGGGTCAA
Ec hmpA	AAGATGCATTTGAGATACATCAA
	TTCTACGTAAACTCTATGTAGTT

*Substitutions giving rise to either predicted repulsive or loss of favorable vdW interactions with *Sc*NsrR (**Table S3**) are in red.

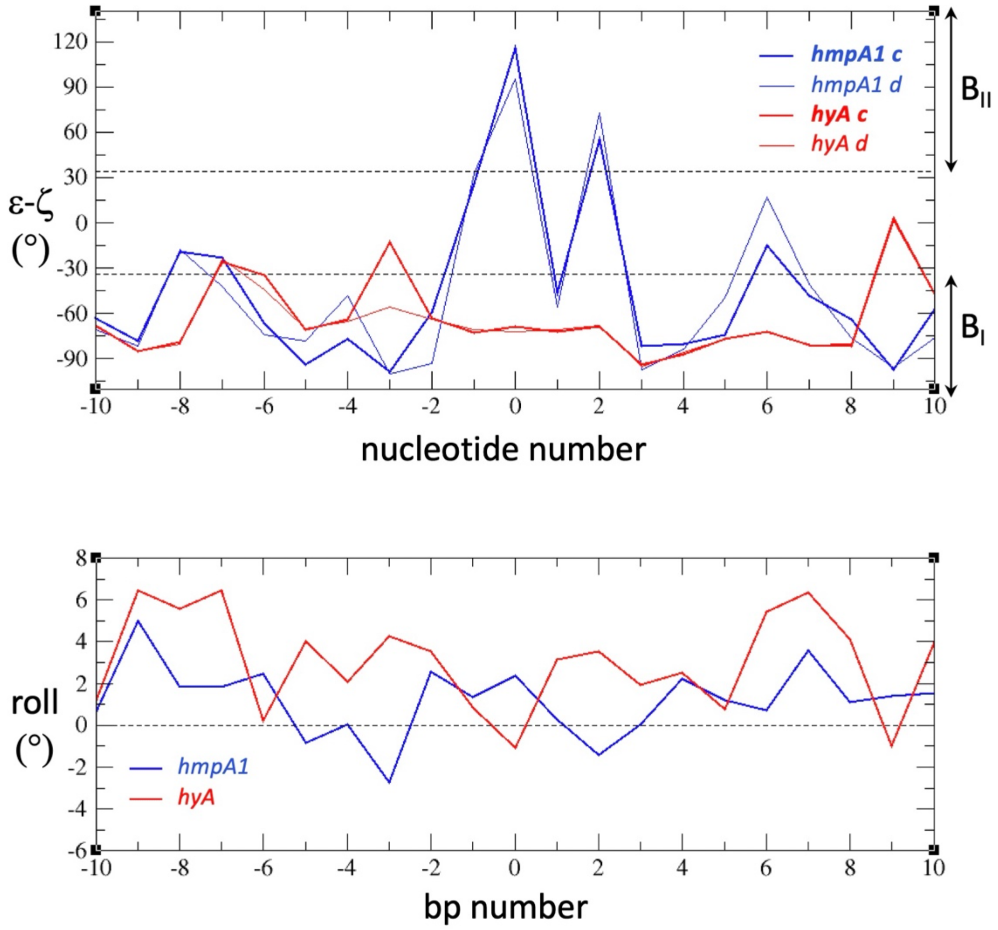

Figure S1. Graphs of ε-ζ and roll angles in the *hmpA1* and *hyA* operators calculated by DSSR[3]. In the ε-ζ graph DNA strands are labeled *c* and *d*.

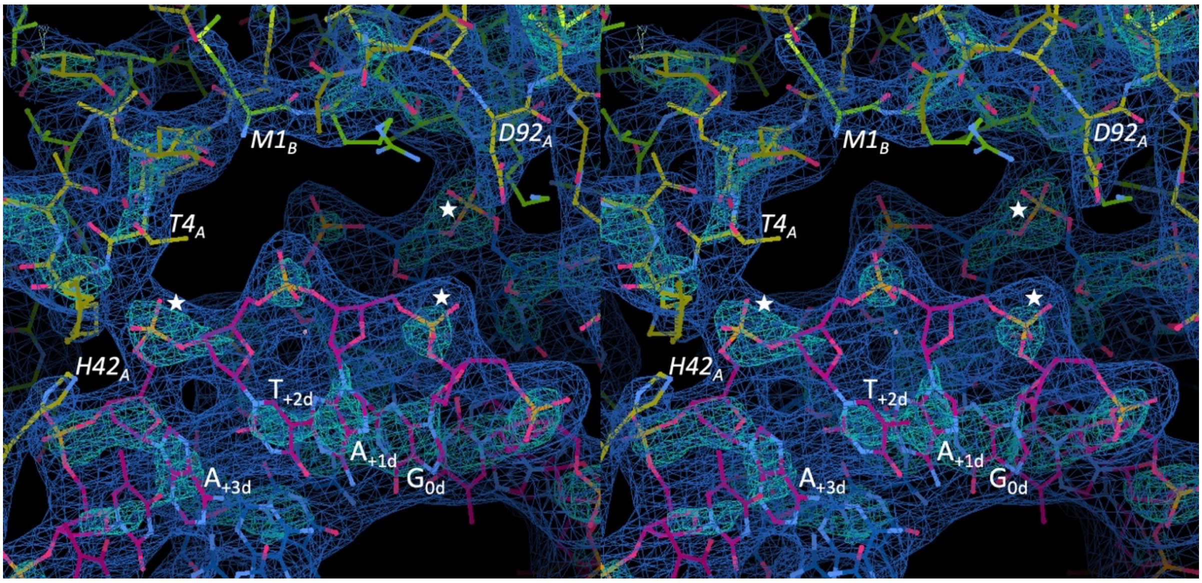

Figure S2. Cross-eyed stereo view of the electron density map around the central region of the *hmpA1* operator. The map is displayed as a grid contoured at 1.0 (dark blue) and 3.0 (light blue) times its root mean square value. Stars indicate B_{II} conformations found with DSSR[3]. Base numbers are given for strand d only, amino acid labels for monomers A and B are given in italics.

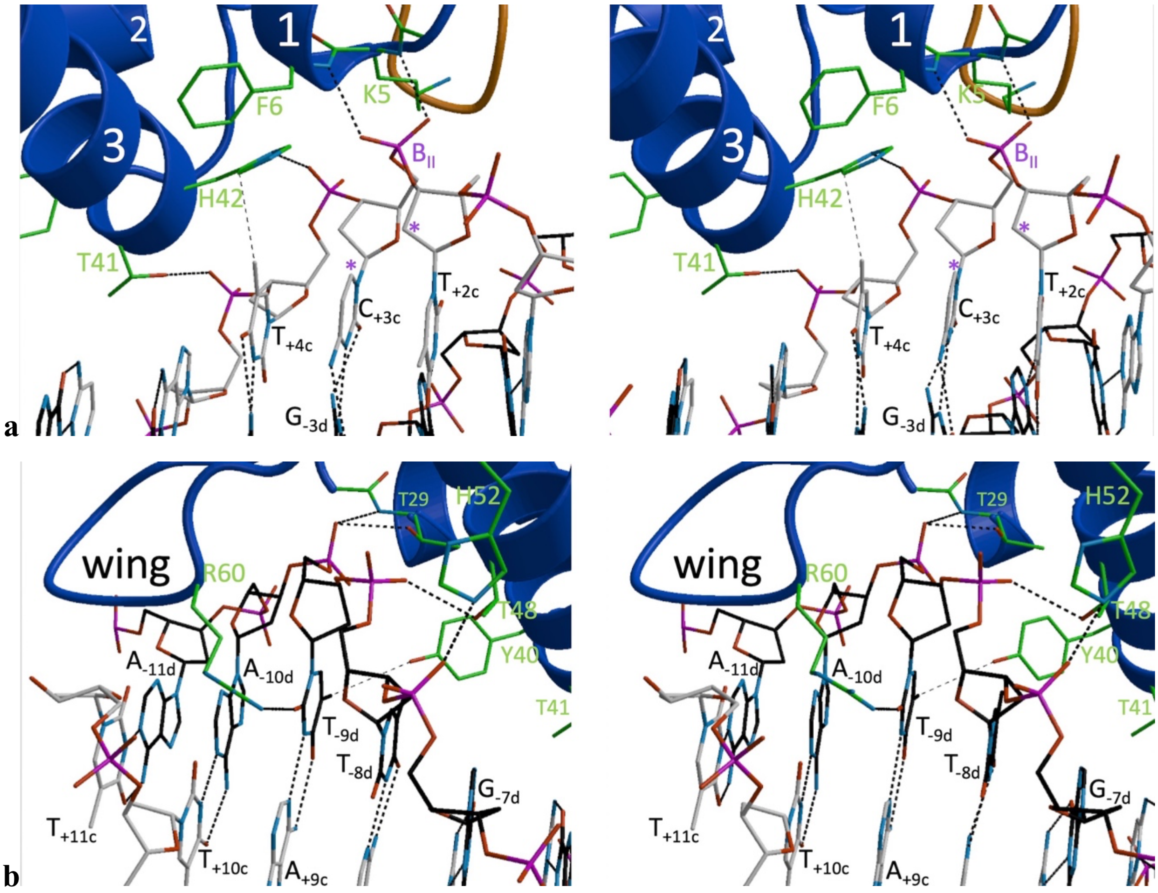

Figure S3. Cross-eyed stereo images corresponding to the right (**a**) and the left (**b**) halves of **Fig. 3.** Asterisks in (**a**) label the C5 and C2' atoms of C_{+3c} and T_{+2c}, respectively. Substitution of the +3 base by T would produce a clash between the C5-bound C7 methyl group and the C2' atom of the +2 base when the connecting phosphate group is in the B_{II} conformation observed for the *hmpA1* operator.

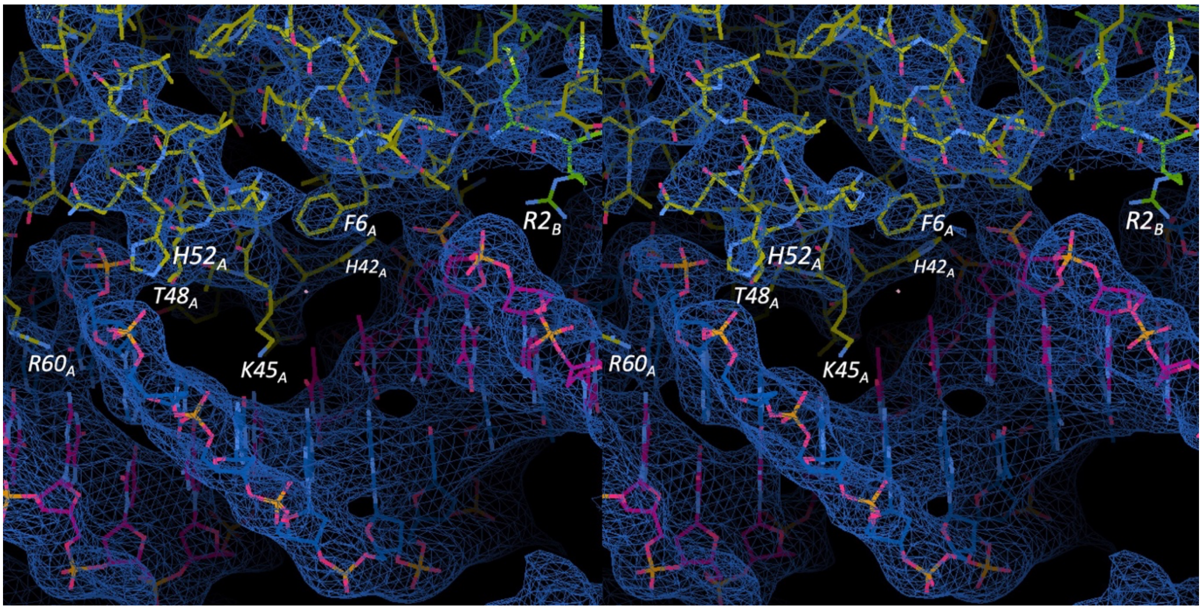

Figure S4. Cross-eyed stereo view of the contact interface between NsrR monomer B and the bound *hmpA1* operator. The electron density map is displayed as a blue grid contoured at 0.75 times its root mean square value. Atom color codes: S yellow, P orange, O red, N cyan and C green. Arg2 and Lys45 are disordered.

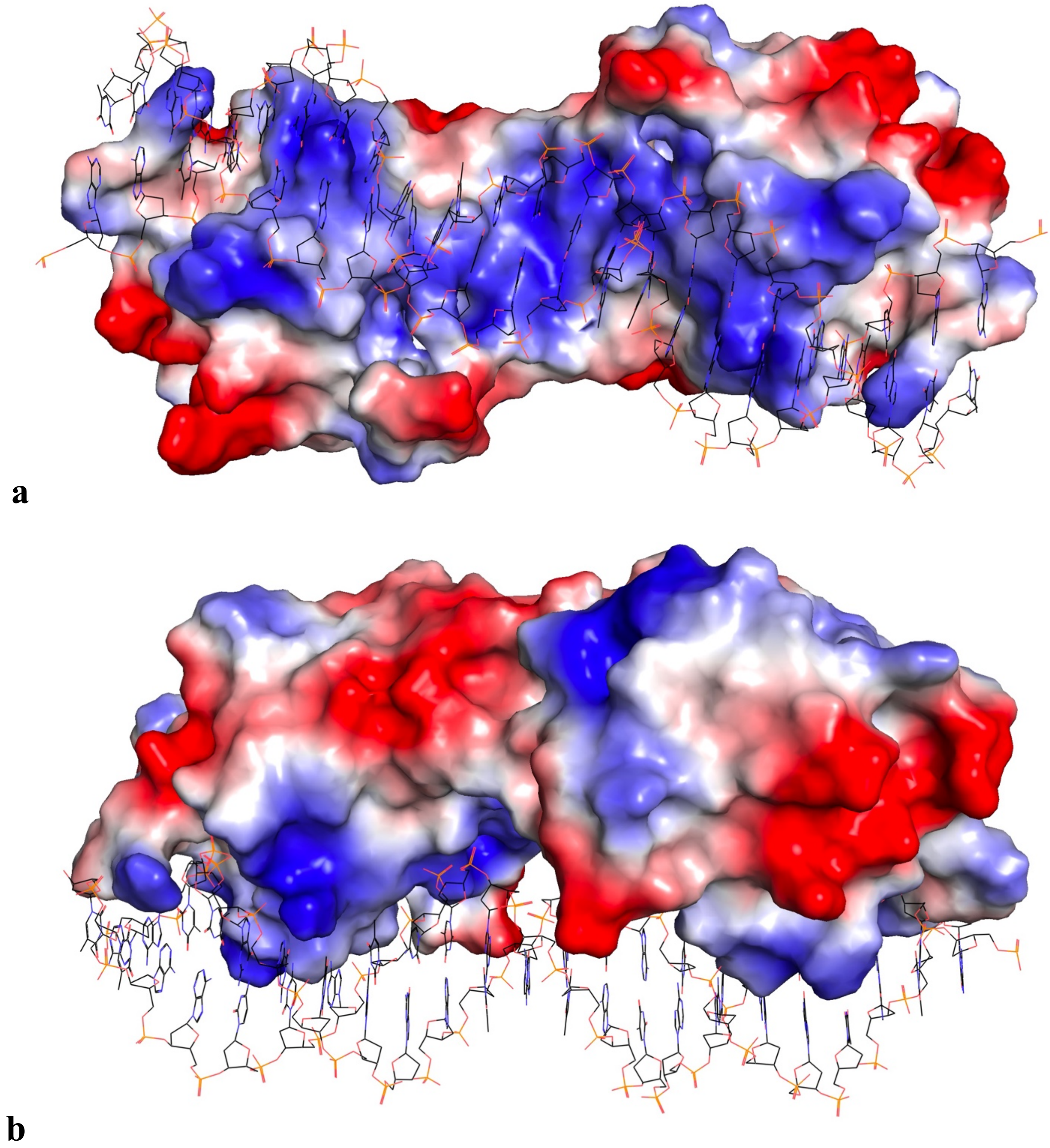

Figure S5. Charge and shape complementarity of *Sc*NsrR and the *hmpA1* operator, (**a**) top view and (**b**) side view. The protein surface is colored according to the local protein contact potential calculated for vacuum electrostatics, with blue and red regions corresponding to positive and negative potentials, repectively. Chemical bonds in the operator double helix are represented with sticks that are color-coded according to atom type. This figure was prepared with PyMOL (www.pymol.org, the PyMOL molecular graphics system, version 2.1 Schrödinger, LLC).

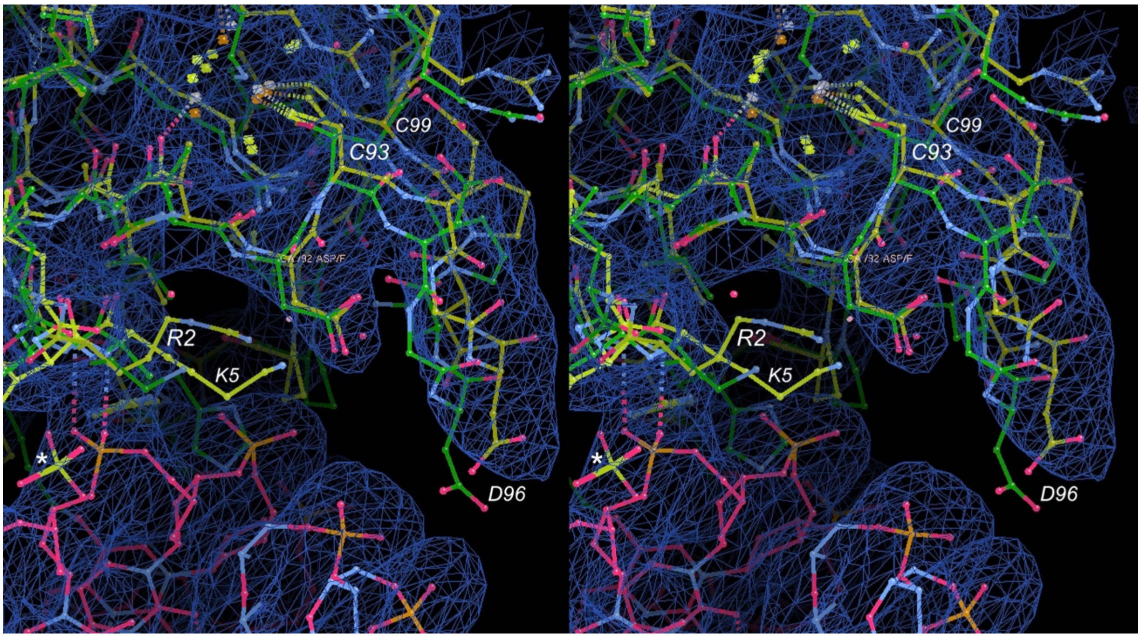

Figure S6. Cross-eyed stereo zoom on the Cys93-Cys99 loop region in a superposition of non-complexed [4Fe-4S]-*Sc*NsrR, with green C atoms to its *hmpA1* operator complex shown with yellow and pink C atoms. Arg2 and Lys5 are disordered in both structures. A sulfate ion in the non-complexed [4Fe-4S]-*Sc*NsrR structure is labeled with an asterisk. The electron density map is displayed as in **Fig. S4.**

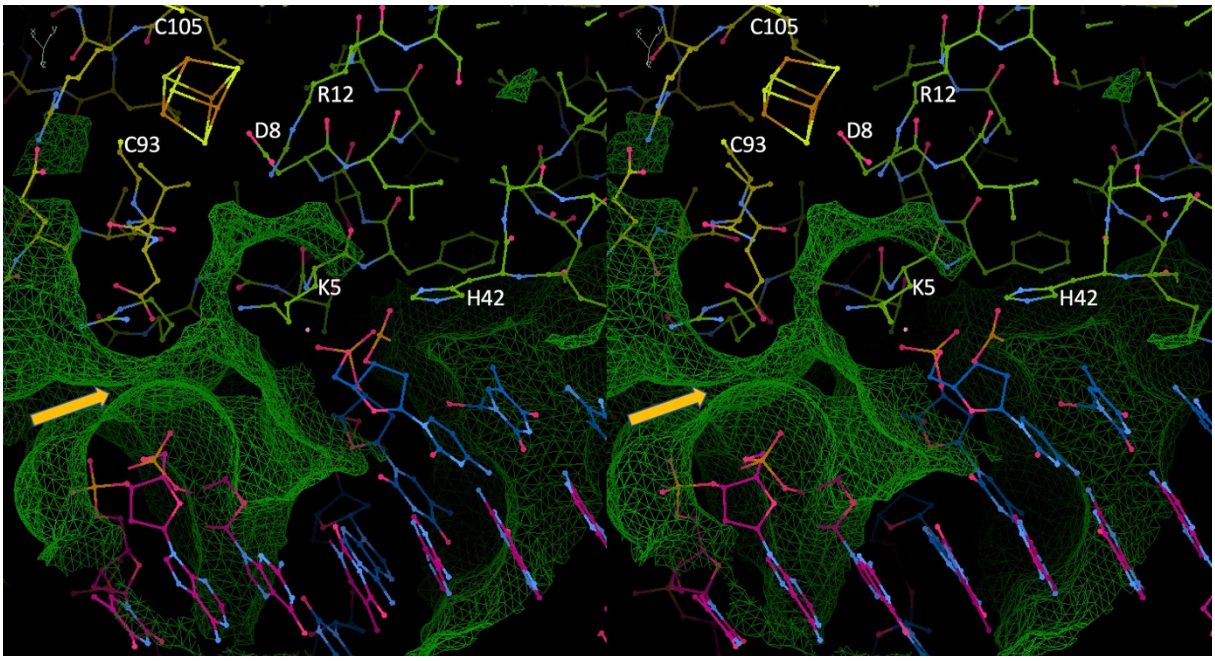

Figure S7. Cross-eyed stereo zoom of a cavity map of the [4Fe-4S]-*Sc*NsrR/*hmpA1* complex. The map is contoured at a probe radius of 1.2 Å and shows that the minor groove of the DNA fragment faces a putative NO access path to the cluster that is connected to the surface, as indicated by the yellow arrow. A similar pathway next to the Asp8 cluster ligand has been found in the non-complexed protein structure.[4] The cavity map was calculated with the in house program CavEnv as included in the CCP4 software package[5].

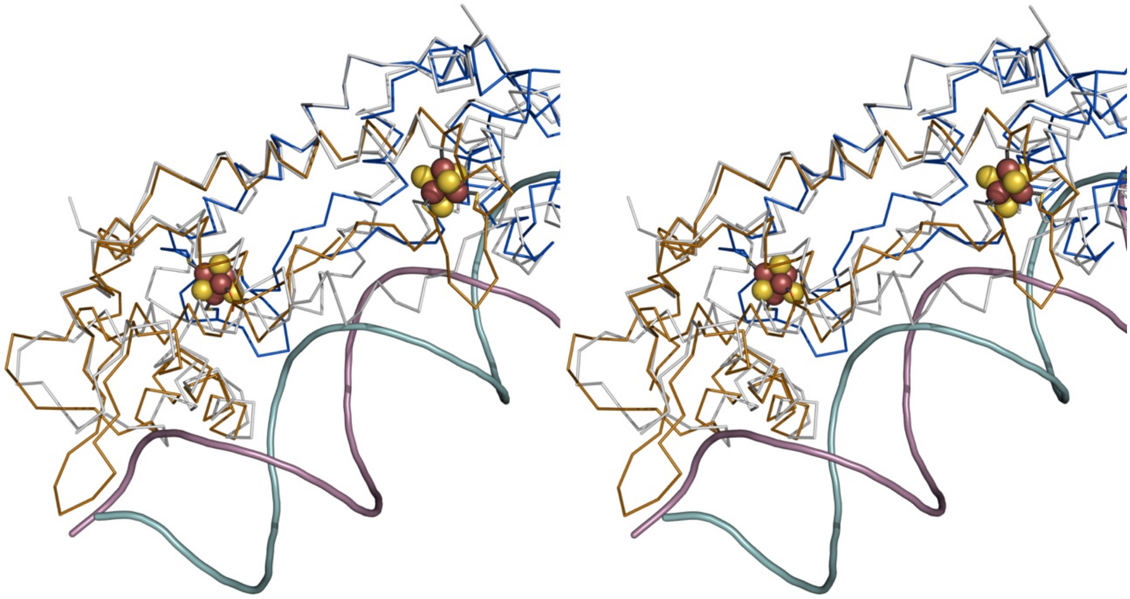

Figure S8. Cross-eyed stereo view of the superposition of apo-3CA-*Sc*NsrR (grey Cα-trace, wing not resolved) on to the [4Fe-4S]-*Sc*NsrR/*hmpA1* operator complex (blue and gold Cα's).

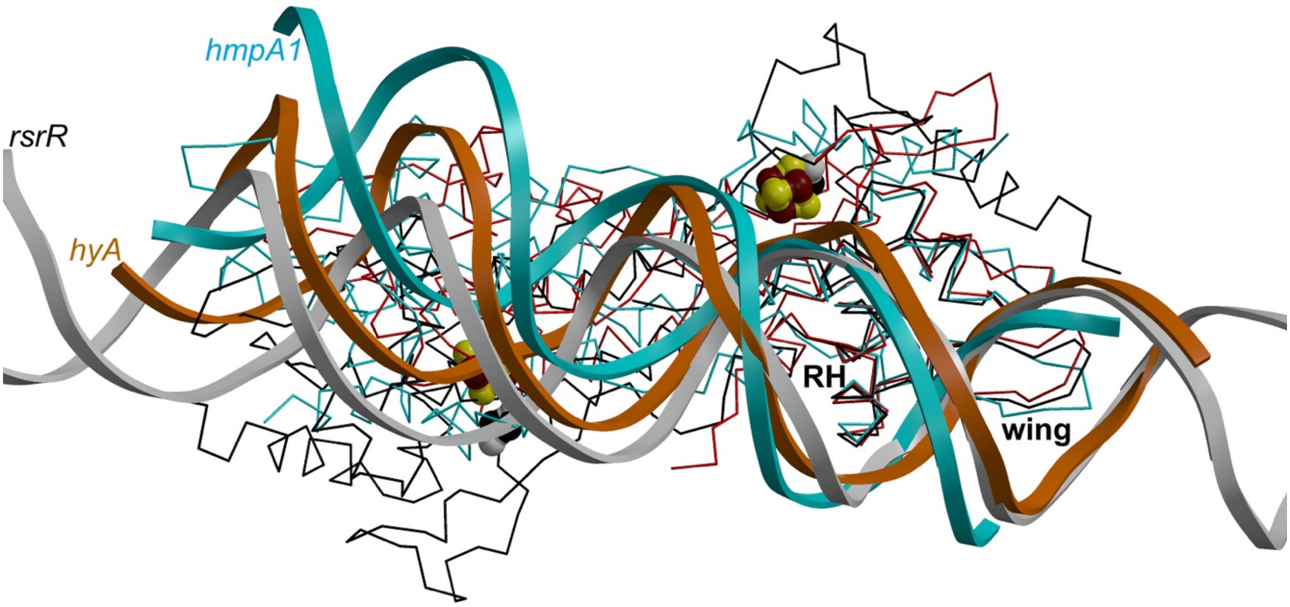

Figure S9. Comparison of dimers of [4Fe-4S]-*Sc*NsrR (cyan), apo-*Ec*IscR (orange) and [2Fe-2S]-*Sv*RsrR (black). The proteins are shown as Cα traces after the superposition of the H-wHTH motifs of one subunit for which the regulatory helix (RH) and wing are labeled. The double helix backbone is traced for the bound labeled DNA fragments, showing *hmpA1* (23 bp) in cyan, *hyA* (29 bp) in orange and *rsrR* (39 bp) in gray.

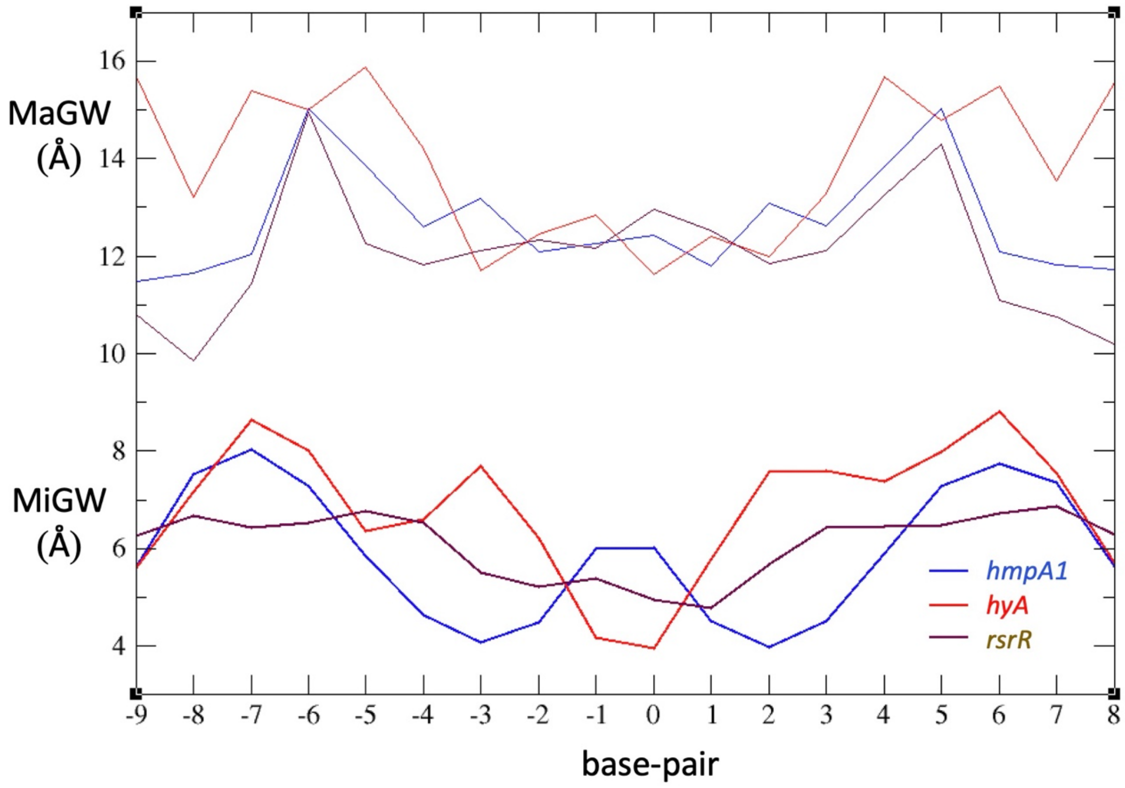

Figure S10. Graphs of minimal (MiGW) and maximal groove width (MaGW) for *hmpA1*, *hyA* and *rsrR*. These are measured with DSSR[3] as MiGW = $0.5[(P_{k+1}-p_{2-k})+(P_{k+2}-p_{1-k})] - 5.8$ and MaGW = $P_{k-2}-p_{-k-2} - 5.8$ according to the method of El Hassan & Calladine[6] (P and p refer to phosphate positions in the two complementary DNA strands and k corresponds to the base number of the top strand in Figs. 2, S1 and S12).

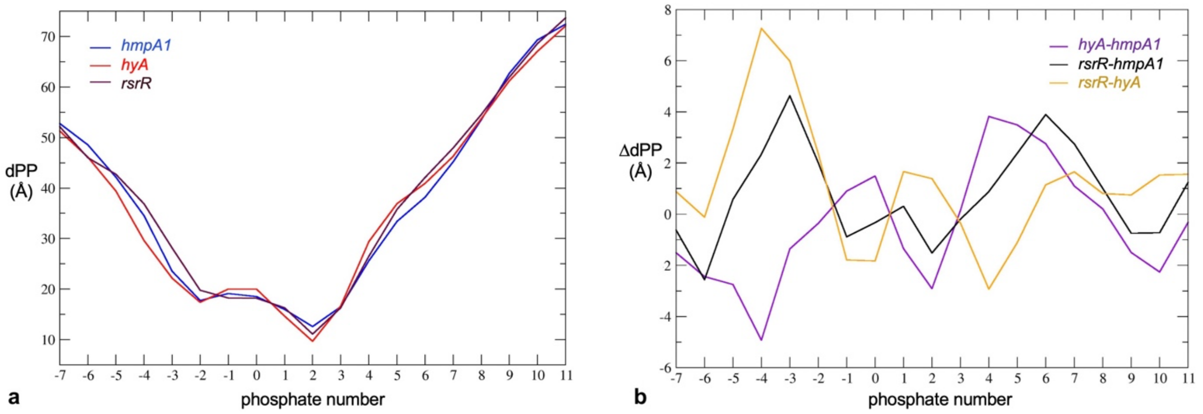

Figure S11. P-to-P distances between pseudo two-fold symmetry-related phosphate groups in the partially palindromic DNA strands ***c*** and ***d*** (**a**) and subtraction of the corresponding dPP values for the *hmpA1*, *hyA* and *rsrR* operator fragments (**b**). These ΔdPP values shows quite significant differences between the three DNA structures, explaining their different binding specificities to [4Fe-4S]-*Sc*NsrR, apo-*Ec*IscR and [2Fe-2S]-*Sv*RsrR. The +4 and +5 phosphates in *hmpA1* are involved in specific H-bonding or salt bridge interactions with [4Fe-4S]-*Sc*NsrR whereas the +2 phosphate in *hyA* is involved in a specific salt bridge with apo-*Ec*IscR (Table S1).

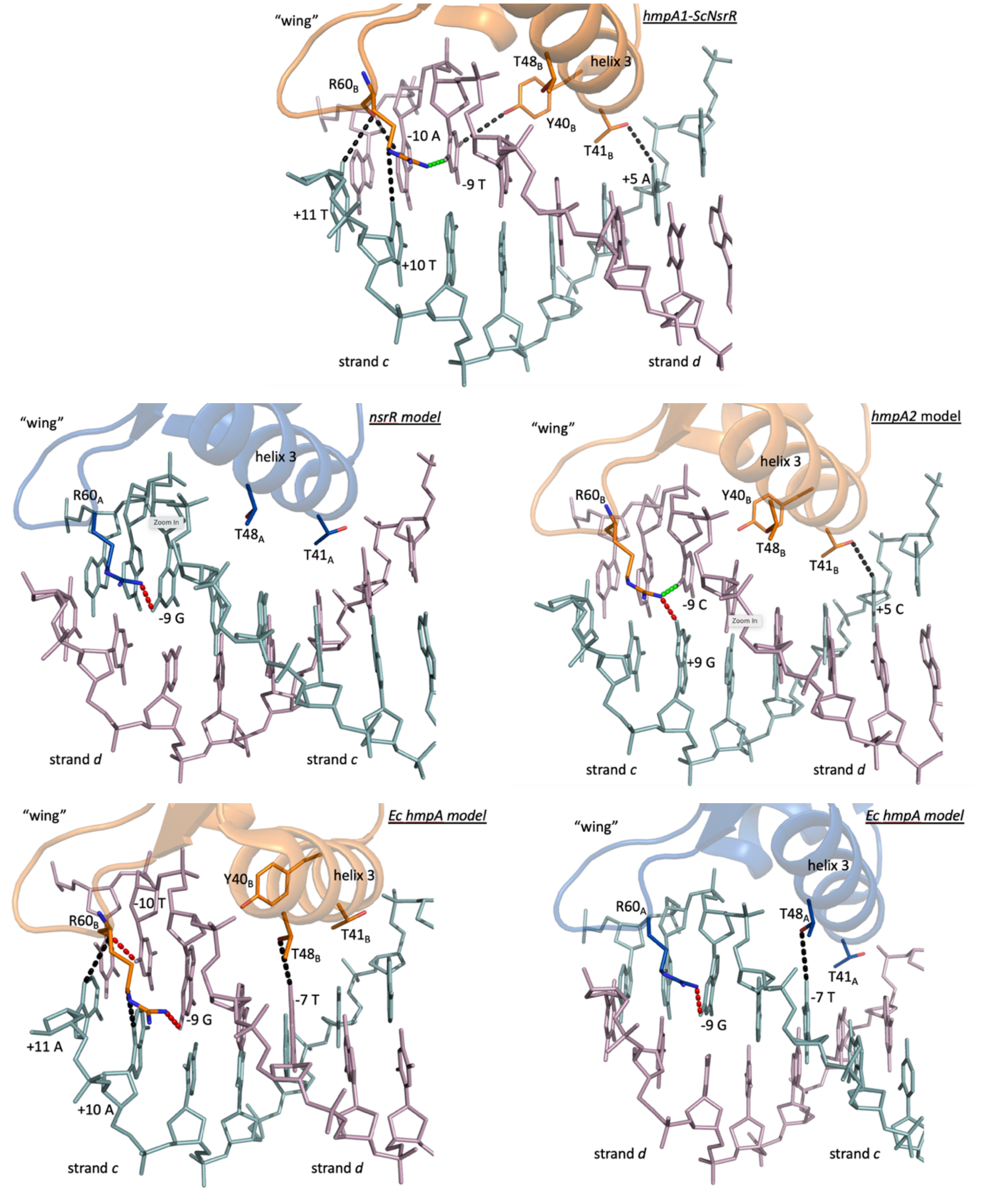

Figure S12. NsrR *hmpA1* base specificity versus *nsrR*, *hmpA2* and (*Ec*) *hmpA* models. The NsrR main chain is drawn as ribbons and strings and selected residues are depicted as sticks; subunit A is in blue and subunit B in orange. The DNA strands *c* and *d* are shown as cyan and burgundy sticks, respectively. Base specific interactions for non-H atoms at distances <4 Å are shown as black dashes, repulsive interactions with distances < 2.85 Å as red dashes, while stabilizing vdW interactions are in green. In the models of *hmpA2*, *nsrR* and *(Ec) hmpA* only the contacts that are not shared with the structure of the *hmpA1/ScNsrR* complex are shown. The latter are modeled on the known structure of the *hmpA1/Sc*NsrR complex by substituting the bases of the *hmpA1* operator for bases matching the sequence of *hmpA2*, *nsrR* and *(Ec) hmpA*, respectively.

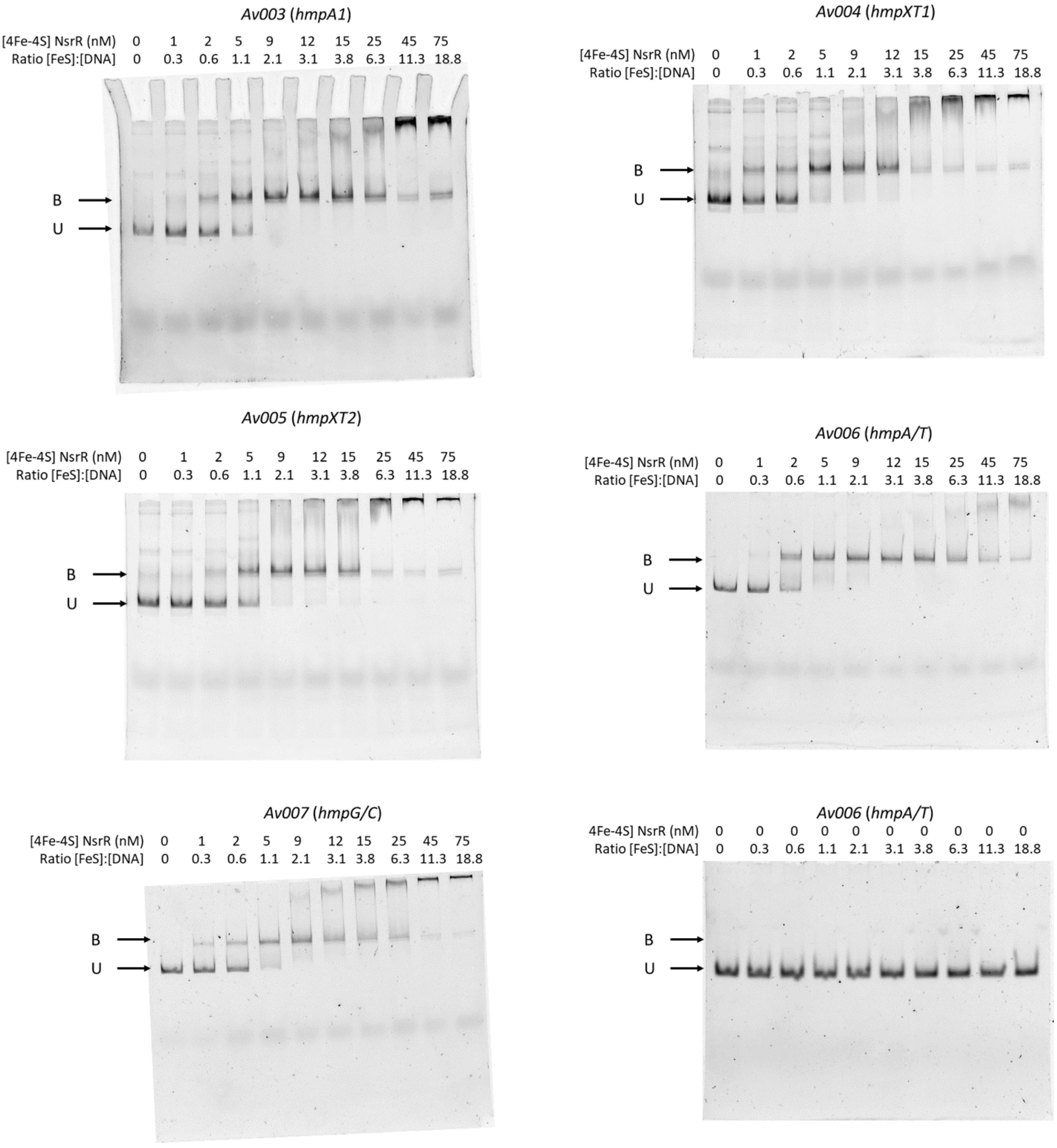

Figure S13. Non-cropped images for the EMSAs shown in Fig. 7. EMSAs show probes (Av003 – Av007) unbound (U) or bound (B) by [4Fe-4S] NsrR.

Supplementary references

1. Crack, J. C. *et al.* NsrR from *Streptomyces coelicolor* is a nitric oxide-sensing [4Fe-4S] cluster protein with a specialized regulatory function. *J. Biol. Chem.* **290**, 12689–12704 (2015).
2. Bodenmiller, D. M. & Spiro, S. The yjeB (nsrR) gene of *Escherichia coli* encodes a nitric oxide-sensitive transcriptional regulator. *J. Bacteriol.* **188**, 874–881 (2006).
3. Lu, X.-J., Bussemaker, H. J. & Olson, W. K. DSSR: an integrated software tool for dissecting the spatial structure of RNA. *Nucleic Acids Res.* **43**, e142 (2015).
4. Volbeda, A. *et al.* Crystal structures of the NO sensor NsrR reveal how its iron-sulfur cluster modulates DNA binding. *Nat. Commun.* **8**, 15052 (2017).
5. Winn, M. D. *et al.* Overview of the CCP4 suite and current developments. *Acta Crystallogr. D Biol. Crystallogr.* **67**, 235–242 (2011).
6. El Hassan, M. A. & Calladine, C. R. Two distinct modes of protein-induced bending in DNA. *J. Mol. Biol.* **282**, 331–343 (1998).
