# Supplementary material for: Structural determinants of DNA recognition by the NO sensor NsrR and related Rrf2-type [FeS]-transcription factors
Source: Commun Biol. 2022 Jul 30;5:769. doi: 10.1038/s42003-022-03745-7 (PMC9338935; doi:10.1038/s42003-022-03745-7)
Supplement: Supplementary file 2 — Reporting Summary [file 42003_2022_3745_MOESM2_ESM.pdf]

nature research

Corresponding author(s): Anne Volbeda

Last updated by author(s): Jun 12, 2022

Reporting Summary

Nature Research wishes to improve the reproducibility of the work that we publish. This form provides structure for consistency and transparency in reporting. For further information on Nature Research policies, see our Editorial Policies and the Editorial Policy Checklist.

Statistics

For all statistical analyses, confirm that the following items are present in the figure legend, table legend, main text, or Methods section.

n/a	Confirmed	
☐	☒	The exact sample size (*n*) for each experimental group/condition, given as a discrete number and unit of measurement
☐	☒	A statement on whether measurements were taken from distinct samples or whether the same sample was measured repeatedly
☐	☒	The statistical test(s) used AND whether they are one- or two-sided<br>*Only common tests should be described solely by name; describe more complex techniques in the Methods section.*
☒	☐	A description of all covariates tested
☒	☐	A description of any assumptions or corrections, such as tests of normality and adjustment for multiple comparisons
☒	☐	A full description of the statistical parameters including central tendency (e.g. means) or other basic estimates (e.g. regression coefficient) AND variation (e.g. standard deviation) or associated estimates of uncertainty (e.g. confidence intervals)
☒	☐	For null hypothesis testing, the test statistic (e.g. *F*, *t*, *r*) with confidence intervals, effect sizes, degrees of freedom and *P* value noted<br>*Give P values as exact values whenever suitable.*
☒	☐	For Bayesian analysis, information on the choice of priors and Markov chain Monte Carlo settings
☒	☐	For hierarchical and complex designs, identification of the appropriate level for tests and full reporting of outcomes
☒	☐	Estimates of effect sizes (e.g. Cohen's *d*, Pearson's *r*), indicating how they were calculated

Our web collection on statistics for biologists contains articles on many of the points above.

Software and code

Policy information about availability of computer code

Data collection	The DA+ software (reference 56 in the main paper) was used to collect X-ray diffraction data at beamline X06SA of the Swiss Light Source.
Data analysis	XDS version Jan 31 2020, CCP4 version 7.1.003, AIMLESS version 0.7.4, PHASER version 2.8.3, Refmac5 version 5.8.0258, PHENIX version 1.18.2, PISA version 2.1.1, DSSR v1.9.9. Compilations of all these programs were provided by SBGrid (ref. 69).

For manuscripts utilizing custom algorithms or software that are central to the research but not yet described in published literature, software must be made available to editors and reviewers. We strongly encourage code deposition in a community repository (e.g. GitHub). See the Nature Research guidelines for submitting code & software for further information.

Data

Policy information about availability of data

All manuscripts must include a data availability statement. This statement should provide the following information, where applicable:

- Accession codes, unique identifiers, or web links for publicly available datasets
- A list of figures that have associated raw data
- A description of any restrictions on data availability

Atomic coordinates and structure factors for the reported crystal structure have been deposited with the Protein Data bank under accession number 7B0C. The two other structures used in our paper are available in the Protein Data Bank under accession codes 4HF1 (ref. 17) and 6Y42 (ref. 21), as given in Fig. 5.

Field-specific reporting

Please select the one below that is the best fit for your research. If you are not sure, read the appropriate sections before making your selection.

☒ Life sciences ☐ Behavioural & social sciences ☐ Ecological, evolutionary & environmental sciences

For a reference copy of the document with all sections, see nature.com/documents/nr-reporting-summary-flat.pdf

Life sciences study design

All studies must disclose on these points even when the disclosure is negative.

Sample size	A single crystal was used to collect all the used X-ray diffraction data (see Table 1)
Data exclusions	All measured data were used for the structure determination
Replication	The resulting structure was confirmed by independently collected data from other crystals. Only the highest resolution structure is reported.
Randomization	A randomly selected subset of diffraction data (5%) were excluded from the refinement of the structure in order to obtain a free R factor.
Blinding	As far as we know, this is not relevant for our study.

Reporting for specific materials, systems and methods

We require information from authors about some types of materials, experimental systems and methods used in many studies. Here, indicate whether each material, system or method listed is relevant to your study. If you are not sure if a list item applies to your research, read the appropriate section before selecting a response.

Materials & experimental systems

n/a	Involved in the study
☒	☐ Antibodies
☒	☐ Eukaryotic cell lines
☒	☐ Palaeontology and archaeology
☒	☐ Animals and other organisms
☒	☐ Human research participants
☒	☐ Clinical data
☒	☐ Dual use research of concern

Methods

n/a	Involved in the study
☒	☐ ChIP-seq
☒	☐ Flow cytometry
☒	☐ MRI-based neuroimaging
